# Heterogeneous interventions reduce the spread of COVID-19 in simulations on real mobility data

**DOI:** 10.1038/s41598-021-87034-z

**Published:** 2021-04-08

**Authors:** Haotian Wang, Abhirup Ghosh, Jiaxin Ding, Rik Sarkar, Jie Gao

**Affiliations:** 1 Department of Computer Science, Rutgers University, Piscataway, USA; 2 Department of Computer Science and Technology, University of Cambridge, Cambridge, UK; 3 John Hopcroft Center for Computer Science, Shanghai Jiao Tong University, Minhang, China; 4 School of Informatics, University of Edinburgh, Edinburgh, UK

**Keywords:** Computer science, Lifestyle modification

## Abstract

Major interventions have been introduced worldwide to slow down the spread of the SARS-CoV-2 virus. Large scale lockdown of human movements are effective in reducing the spread, but they come at a cost of significantly limited societal functions. We show that natural human movements are statistically diverse, and the spread of the disease is significantly influenced by a small group of active individuals and gathering venues. We find that interventions focused on these most mobile individuals and popular venues reduce both the peak infection rate and the total infected population while retaining high social activity levels. These trends are seen consistently in simulations with real human mobility data of different scales, resolutions, and modalities from multiple cities across the world. The observation implies that compared to broad sweeping interventions, more heterogeneous strategies that are targeted based on the *network effects* in human mobility provide a better balance between pandemic control and regular social activities.

## Introduction

The global pandemic of a novel coronavirus (SARS-CoV-2) has swept through nearly all countries since December 2019. Due to the high infection rate and therefore high demand for medical resources, most countries have adopted large-scale interventions such as business lockdowns and restricted movements. These intervention methods have proven to be effective—successfully reducing the number of peak daily infected cases and the total number of infected cases so far, as shown by both direct observation from real data^1,2^ as well as indirect metapopulation models and simulations^3,4^.

While these intervention methods have helped in controlling the pandemic, they are not sustainable over long periods. To address this challenge, we must understand the effect of human movement, and if a less disruptive intervention strategy can control the epidemic.

The relation between mobility and spread of COVID-19 has been observed in several recent studies^4–6^ that show empirical evidence of correlation between aggregate mobility and number of cases. These studies do not consider the social costs^7^ (for example, the reduction in social and economic activities) and are at large scales that do not provide the understanding necessary for more targeted interventions^2^. Classical epidemiology approaches can model behaviour at scale of individual agents—these include models such as SIR or SEIR^8^ and their variations^3,9,10^, data-driven models^11^, and multi-agent models^12^. By amending the epidemiological models with characteristics of COVID-19 and incorporating the effect of real human movements, we can gain insights into targeted strategies for interventions and the social costs of interventions.

The objective of our study is to understand the effect of human movements in the spread of the virus. We use an agent-based simulation where a mobile agent can probabilistically infect other agents that are in proximity—such as at the same venue. We consider movements of the real people captured in three types of mobility data—check-ins at seven different cities, WiFi connection events on a university campus, and GPS traces of electric bikes—representing different scales, behaviors, and modalities. Across these diverse circumstances, we observe several common features in human movement, the spread of the epidemic, and the effect of interventions.

## Results

**Figure 1:**
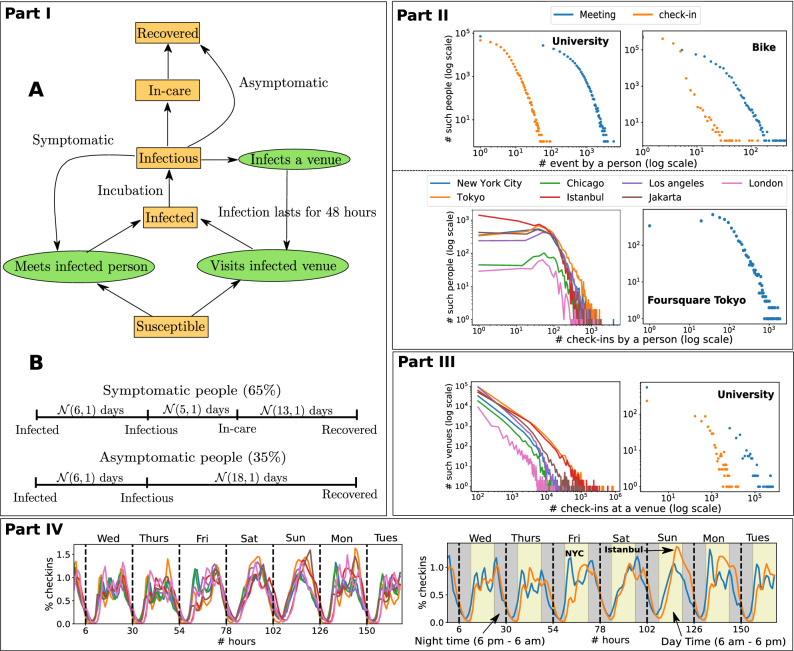
The model for infection spread (Part I) and dataset properties (Part II to IV). The model (**A**) is adapted from SEIR infection spread model and the parameters (**B**) are from CDC for COVID-19. The model states and actions are represented by boxes and ovals respectively. Part II and III present distributions of the number of check-ins or meetings per person and per venue, depicting the heterogeneity of mobility. Part IV: Normalized check-in counts aggregated over a week for all Foursquare datasets show daily patterns with increased activity on weekends. Patterns vary across cities.

**Figure 2:**
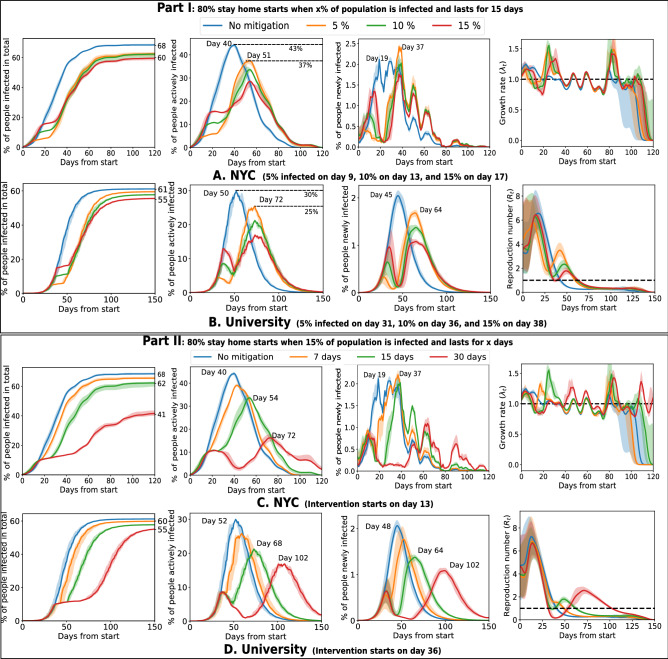
Uniform lockdown for limited duration delays and lowers the peak of the infections, but often has a small effect on total infection. Here, $80\%$ check-in or meeting events are randomly removed. Part I: A limited duration intervention (15 days) delays (by 11 days in NYC and 22 days in the University) and lowers (by $6\%$ in Foursquare NYC and $5\%$ in the University) the peak of active cases, but does not reduce the total number of infected people, as the second peak is imminent. Part II: With the increasing length of intervention, the number of infected people reduces (by $33\%$ in NYC), but in the University dataset, the effect is milder.

**Figure 3:**
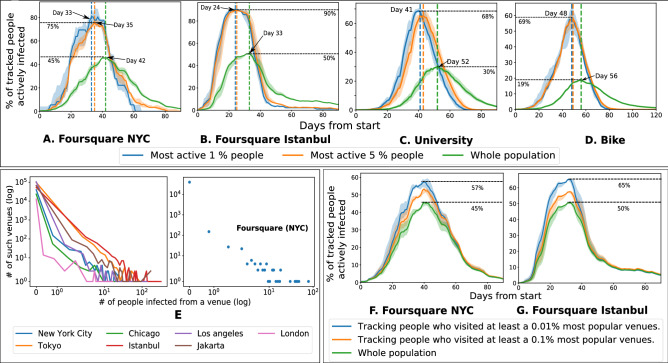
Heterogeneity of mobility in venues and agents results in varied risks to catch and spread the virus. Agents with higher activity get infected with a higher proportion and earlier (**A**–**D**). In all Foursquare datasets, the number of people infected from a venue has heavy-tailed distributions (**E**). People who visit the most popular venues get infected in higher proportions (**F**,**G**).

**Figure 4:**
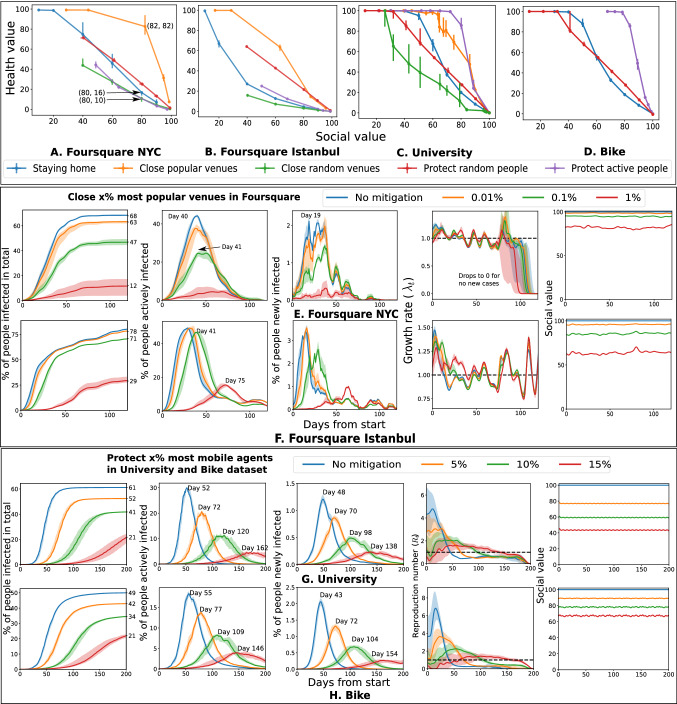
Across all datasets, the most effective strategies in terms of their health and social values consider the heterogeneity of mobility (**A**–**D**) and (**E**–**H**) present the details of the best strategies. The health value quantifies the percentage of people not infected compared to without intervention scenario, and the social value is the percent of check-ins or meetings that remain under intervention. An ideal strategy would have both high health, and social values. Across Foursquare datasets, closing the most popular venues is the most effective strategy (**A**,**B**); isolating or protecting the most active agents is the best strategy in University (**C**) and the Bike (**D**) datasets. The Bike dataset does not have venues and therefore it omits corresponding strategies. (**E**)–(**H**) show details of the best strategies. They reduce infections to a large extent (closing the most popular $1\%$ venues results in $57\%$ reduction in infection in the NYC dataset), delays and lowers the peak of active infections (protecting the most active $5\%$ agents in the University dataset delays the peak by 20 days) and maintain high social values ($1\%$ most popular venues contribute to $20\%$ check-ins in the NYC dataset).

**Figure 5:**
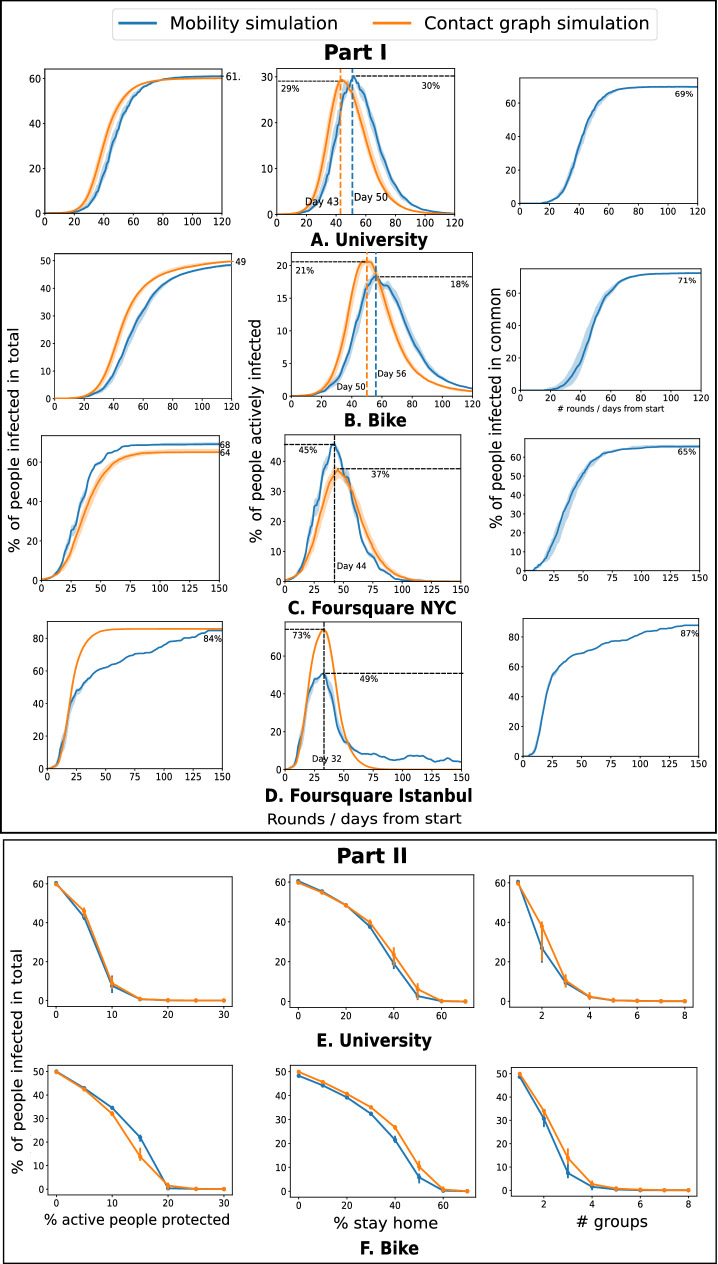
Contagion simulation in the derived social network model infects a similar number and similar set of agents as the simulation using mobility data in both with and without intervention settings. Part I: The curves for total infection numbers, actively infected agents, and Jaccard similarity of the infected agents match under no intervention scenario (**A**–**C**). Although in the Istanbul dataset (**D**), two infection number curves do not match after 25 days, similar sets of people get infected in the two simulations. Part II: In person to person infection spread model, with different intervention parameters, the final infection number is similar between the two models in all three intervention strategies.

We found that mobility statistics are heterogeneous across individuals and venues (Fig. 1), with few agents being highly active and some venues attracting many visitors. This heterogeneity and consequent network effects influence the spread of COVID-19. The common strategy of blanket lockdown is seen to delay the peak but comes at a high social cost (Fig. 2). High mobility agents and popular venues attract early infections and play an important role in the early spread of the infection (Fig. 3). An important observation from our simulations is that interventions that protect these active entities, such as isolating or immunizing the highly active agents, or closing the popular venues, are seen to have a large effect—both delaying the peak and reducing total infections—while still incurring a relatively small social cost (Fig. 4). Classical models (e.g.SEIR^8^) where infection transmission is not related to spatial proximity do not show such heterogeneous effects (Supplementary Fig. S7), which implies that mobility makes a significant difference compared to homogeneous models. Finally, a social network or *contact graph*—that connects agents to other agents they meet frequently or to venues that they visit frequently—is seen to have the property that a similar number and group of people are infected in this static model as under the dynamic mobility simulation model (Fig. 5). This section elaborates on these results, starting with a study of the baseline lockdown strategy.

### Evaluation of baseline uniform mobility intervention strategy

Under a lockdown or stay-at-home order, mobility drops only to a limited extent^13^. We model such a partial lockdown by randomly removing events with a certain probability. This is our baseline intervention strategy. Figure 2 studies the realistic scenario where the intervention lasts for a limited duration, with $80\%$ of the events removed. Thus, during intervention this retains $20\%$ social value (experiments with other probabilities are in Supplementary Fig. S1 online). The results agree with the observations across the world—the peak of the infection is lowered and delayed^2^. However, we discover that this strategy alone does not significantly reduce the total number of infections when applied for a limited duration. Supplementary Fig. S2 shows the effects of such a strategy applied for longer durations.

Part I of Fig. 2 studies the disease spread with a varied starting time of the intervention while keeping its duration fixed at 15 days. In the Foursquare NYC dataset, the intervention starts on the day 9 when $5\%$ of the population gets infected. Compared to the scenario without intervention, this strategy reduces the total number of infected agents by $8\%$, delays the peak of active infections by 11 days, and lowers it by $6\%$. The peak of new infections is also delayed by 18 days. The University dataset shows a similar pattern, as the peaks in active and new infection are delayed by 22 and 19 days respectively. Other datasets show trends consistent with the above (Supplementary Fig. S3 online).

We note that an earlier lockdown is not necessarily the better strategy when implemented for a fixed duration. An imperfect lockdown can allow the infection to survive, which then spreads rapidly on the removal of the intervention and produces a high eventual peak or second wave. On the other hand, a lockdown implemented somewhat later achieves a lower peak later on, and a lower total number of infections.

In Fig. 2, Part II, the intervention strategy starts when $10\%$ of the population is infected (day 13 for the Foursquare NYC dataset and day 36 for the University dataset). The longer interventions have a more significant effect in reducing both the peak of active cases and total infections. A 30-days intervention reduces the total infected population by $27\%$ and $5\%$ in the Foursquare NYC dataset, the University dataset respectively. While for shorter interventions (up to 15 days), the active infection curves remain uni-modal, a longer intervention (30 days) produces two peaks and the second peak is delayed by 32 and 65 days for the Foursquare NYC and the University dataset respectively. The growth rate, $\lambda _{t}$ for the longer intervention does not go to zero ($\lambda _{t}$ reaches zero when no new person is infected). Thus the disease remains in circulation for a longer period. Interventions up to 2 months (Supplementary Fig. S4 online, Part II) show similar conclusions: second peaks are observed, and the cumulative number of infected people does not decrease beyond $10\%$ compared to the setting without intervention.

**Figure 6:**
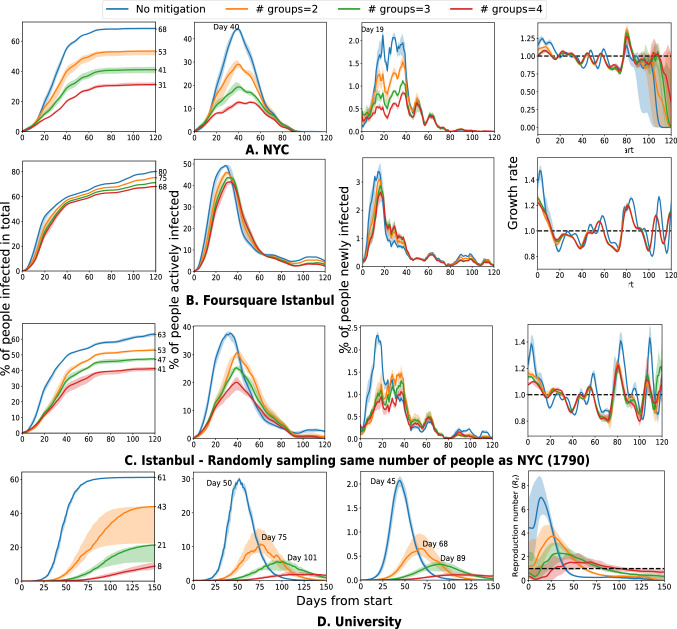
Diving agents into cohorts cuts off the contacts between different cohorts, like separating students into different sessions for courses and acts to reduce the spread of the disease. With the more groups, the number of infected people is reduced (by $37\%$ in NYC and by $53\%$ in the university with 4 groups). The large population of the Istanbul dataset produces densely connected groups that result in substantial intra-group infection spread (**B**). Thus, when we sample the same number of people as NYC, the effect of separating groups re-appear (**C**). In addition, the peaks of active cases are also delayed and flatten, especially in the University dataset.

Dividing a population into non-interacting cohorts has been proposed as an intervention strategy^14,15^, with the idea that preventing transmission across group boundaries will reduce the spread. We find that grouping does act to delay and lower the peaks and to reduce the total number of infections in Figs. 6 and S5. The number of infected people is reduced by $37\%$ in NYC and by $53\%$ in the university with 4 groups. The effect is milder in the Foursquare datasets with larger population size, for example in the Istanbul dataset (Fig. 6B). By taking the same sized population as in the NYC dataset (Fig. 6C), the effect appears—the total number of infected agents is reduced by $22\%$ with 4 groups.

### Heterogeneity in mobility and contagion dynamics

We found that the most active individuals are infected significantly earlier (Fig. 3) than the average population. A person’s activity is quantified by the number of check-ins in the Foursquare datasets and the number of meetings in the University and Bike datasets. The popularity of a venue is measured by number of check-ins. Of the top $5\%$ most active agents, approximately 60–80% get infected at the peak of active infections compared to the peak height about 20–40% for the overall population, and this peak is reached about 10 days earlier. Thus, highly active people are at a higher risk, and more likely to be propagators of the disease in the early stages. Similar results on other datasets can be seen in Supplementary Fig. S6.

The heterogeneity creates fundamental differences in infection spread compared to the homogeneous SEIR model. Supplementary Fig. S7 shows that in comparison to the homogeneous SEIR model—where agents meet all other agents with equal probability—the data-driven simulation achieves a higher peak, but a smaller total number of infections.

The heterogeneity of venues in spreading the contagion is seen in Fig. 3E–G. The number of agents infected from a venue has a heavy-tailed distribution for all cities in the Foursquare datasets, and the results for the NYC dataset are shown separately. Most of the venues infect a tiny number of individuals, but a few venues infect a large number of agents—In the Foursquare NYC dataset, $50\%$ people are infected from $0.05\%$ (19 venues) most popular venues. There are 718 and 4979 individuals who have visited the top $0.01\%$ most popular venues in the Foursquare NYC and Istanbul datasets respectively. The shape of active infections curve for the active agents remains similar to that for the overall population, but these agents are infected in higher proportions (by at least $12\%$ higher).

### Targeted interventions on the most active individuals and the most popular venues

We study heterogeneous intervention schemes targeted to put a higher level of protection on the most active agents and most popular venues, thus reducing or eliminating their contribution to spreading the virus.

In these simulations, the *social cost* of an intervention is defined as the fraction of social events (or potential interactions) lost due to the intervention. For example, in the check-ins dataset, this is measured as the fraction of check-ins lost. In other datasets, the social cost is measured as the fraction of co-located pairs of individuals—representing potential pairwise meetings. The *social value* is correspondingly measured as the complement of the social cost—that is, the fraction of events preserved under the intervention. The *health value* of an intervention is measured as the fraction of agents who escape infection due to the intervention (but would have been infected otherwise).

Figure 4A–D use the social, and health values to compare the targeted interventions against multiple other strategies including the uniform intervention of staying home, protecting a random subset of agents and closing a random subset of venues. In the Foursquare datasets, closing the most popular venues is the most effective strategy, while protecting the most active agents is the most effective strategy in the University and Bike datasets.

In the NYC dataset, to achieve a similar $\sim \, 80\%$ social value, the strategy of closing the most popular venues shows 60–$72\%$ more health value than other strategies. In the Istanbul dataset, to achieve $60\%$ social value closing the most popular venue achieves at least $52\%$ more health value than other strategies. The other cities in the dataset show consistent patterns (Supplementary Fig. S8 online). In both the University and Bike datasets, at $80\%$ social value, protecting the most active agents achieves $30\%$ or more health value than other strategies.

The strategy to close the most popular venues is analyzed in detail in Fig. 4E,F, and mentary Fig. S9. In Foursquare NYC and Istanbul datasets, closing a few ($\sim \,1\%$) most popular venues reduces the total number of infected individuals by more than $40\%$, the peak number of active infections by $\sim \,30\%$ and delays the time of the peak by up to 30 days. In larger sample populations, this strategy has a smaller impact on total infections and the peak number but serves to delay the peak. In the university dataset, closing $5\%$ of the most popular venues can reduce the total number of infected individuals by $50\%$ and the peak number of active infections by more than $20\%$.

Figure 4G,H, and Supplementary Fig. S10 show the effect of protecting the infection of the most active agents. In the University and Bike datasets, protecting the most active 5–10% of the population reduces total infection by 20–$40\%$ and delays the peak by 90–110 days. Protecting the most active individuals in the Foursquare datasets has similar effects—in the NYC dataset, the total number of infected people is reduced by $\sim \, 30\%$ and the peak of active cases is reduced by $\sim \,25\%$ when $20\%$ most active people are protected. However, protecting $20\%$ most active people reduces the social value by $\sim \, 45\%$. The rest of the Foursquare datasets show similar patterns.

We study other intervention strategies where the agents and venues to protect are selected randomly in Supplementary Figs. S11 and S12. They are used in the comparison of different interventions in Fig. 4.

### Contact graph: social network abstraction to estimate mobility based infection spread

We designed social network models derived from mobility data such that the spread of infection in a mobility dataset resembles that in the corresponding social network. To model person to person disease transmission as a social network, we define a graph *G* with agents as nodes and edges connect agents who have met at least once. Each edge has a weight, *w*, as the average number of times they met in a day. For infection spread via venues, the social network is defined using a bipartite graph where the agents and venues constitute the two sets of nodes, and they connect if a person visited a venue. The edges are weighted by the average number of daily visits.

Figure 5 compares the contagion simulation on a social network against the simulation on time ordered mobility data. The total number of infected people matches between the simulations (Fig. 5, Part I). Further, nearly the same set of agents get infected starting with independent random seed agents. We measure the similarity of the agents infected in two simulations using Jaccard similarity which is defined for sets *A*, *B* as $\frac{|A\cap B|}{|A\cup B|}$. For all datasets, Jaccard similarity between the agents (identified by unique user identifiers in the datasets) infected in the mobility-based simulation and the social network simulation reach above $65\%$. The active cases’ peak differs by $1\%$ (in University) to $24\%$ (in Istanbul). Supplementary Fig. S13 shows similar patterns for other Foursquare datasets.

For the person to person transmission model, Fig. 5, Part II compares the total number of infected people for three intervention strategies: staying home, protecting the most active agents, and dividing people into groups. The strategies are simulated in the social network setting by preprocessing the graph, *G*. The staying home intervention of skipping a meeting with probability $\alpha$ is simulated by removing an edge with weight *w* with probability $1-(1-\alpha )^{w}$. An agent’s activity is defined as the total weight of the edges incident on the corresponding node. The strategy to protect the most active agents translates to removing the nodes with the highest activities from *G*. The strategy to divide the population into groups is simulated by randomly assigning the nodes to groups and then removing the edges between nodes belonging to different groups. The infection curves for mobility and social network simulations are matched for all strategies for both University and Bike datasets—the difference between the two curves is always below $10\%$. The percentage of people infected in total goes to zero in two models with similar intervention parameters.

### Robustness of results

The conclusions from the simulations are robust to parameters changes in the simulation, and has been confirmed in a range of experiments using different parameter values—for infection probability ($\beta$) (Supplementary Fig. S14 online), number of seeds (Supplementary Fig. S15 online), and the probability of an agent being asymptomatic (Supplementary Fig. S16 online). Decreasing $\beta$ reduces the number of infected people and delays the peak of active infections. In the NYC dataset, $68\%$ of the population gets infected when $\beta =0.75$ compared to $34\%$ at $\beta =0.25$. In the Istanbul dataset, the peak is delayed by 10 days when $\beta$ is reduced to 0.25 from 0.75. Increasing the number of *seeds*—initial infected people—increase the robustness or predictability of the spread of infection, but otherwise has a relatively small effect: a moderate number of seeds shows a similar behavior to a larger number of seeds – the total number of infected people differs no more than $4\%$ across datasets for using 20 seeds compared to 1 seed. The fraction of asymptomatic carriers increases the rate of infection (since these carriers remain infectious for a longer period), but does not cause a major growth in infection—in the NYC dataset $14\%$ more people get infected when increasing the percentage of asymptomatic people from 15 to $75\%$. A similar pattern is found in the other datasets.

The conclusions are also sustained when simulations are carried out on sub-samples of data (Supplementary Fig. S17 online). Here a fraction of the agents are randomly sampled for simulation. For the same fraction of the most active agents protected within the subsample, a larger population has a larger fraction of the total population infected. However, this growth appears to be sublinear, and the effect tapers off with the size of the population. Sampling has a similar effect under the intervention of closing popular venues.

## Discussion

We have shown that the diversity of human movement influences the spread of the virus, making the behavior different from homogeneous models. The heterogeneity can be leveraged to devise less disruptive strategies of intervention. Our conclusions are complementary to other non-pharmaceutical interventions such as maintaining person to person distance, wearing masks and avoiding contact. The analysis is based on datasets that are samples of population behavior, and thus, the infection numbers or percentages generated by the simulations should not be regarded as precise representative values but rather as patterns to expect under various interventions. The trends in relative measurements hold across multiple datasets of varying sizes and representing multiple locations, lifestyles, and geographic scales, giving us confidence that they reflect general properties of infection spread due to mobility and corresponding interventions.

Mobility in a region may be affected by policies and interventions applied to other regions such as neighboring districts^16^. This spillover effect can be seen as a network effect on a larger scale. It has been shown^17^ that spillover indeed affects aggregate mobility in US counties and thus intervention policies should be coordinated at larger scales. Our datasets do not include mobility across such administrative zones and consequent spillover effects. As more mobility data from the ongoing pandemic becomes available, it may be possible to incorporate and model such larger-scale network effects on agent mobility and infection, as well as incorporate changes in human behavior due to COVID-19^18,19^. The datasets used here are of pre-COVID behavior between (2012 and 2018) and representative of the spread of infection under “normal” behavior. The effect of changed mobility under social distancing and COVID awareness will require corresponding datasets and separate study.

In simulations, we found that the existing common strategies come with some caveats. The cohort strategy, i.e. partitioning the population into non-interacting groups^14,15,20^ is effective in circumstances such as small populations, a large number of groups, and the specific dataset of University mobility (Fig. 6), but not necessarily in other scenarios. The simulations are restricted to certain slices of the society, and in practice, it is infeasible to maintain a consistent partitioning across different communities such as residence, work, and school, which is likely to reduce the impact of this strategy. A larger number of partitions or cohorts will reduce the spread of infection, but a large number of cohorts are difficult to implement and schedule, and they come at a larger social cost of reduced interaction across cohorts. The implementation of a cohort strategy may differ by circumstances, such as separating the groups spatially (different classrooms) versus separating temporally (different time slots), which will influence the person-to-person distancing and infection propagation. For the other common strategy of a blanket lockdown, which many countries have implemented, the results in Fig. 2 show that the timing and duration of the action subtly influence the peak and total infections. Since this strategy is a limited resource—in the sense that it cannot be sustained for long—it needs to be deployed with careful planning, and with consideration of available medical and economic resources.

We found targeted interventions on the most mobile agents and most popular venues to have a significant effect on pandemic control at a low social cost (Fig. 4). These results are complementary to prioritizing protections for essential workers such as medical professionals^21,22^, as our conclusions are for the mobility component of infection spread, which is not addressed in other works^23^. In this respect, those most at risk and likely spreaders of infection are workers whose professions require frequent movement. While isolating them from work is not practical, better protection strategies such as protective equipment, strict regulations, and early vaccinations can help to reduce infections. Closing the most popular venues such as university cafeteria (Supplementary Fig. S18) are also unsustainable over long durations, but modified operations and strict social distancing at these venues can help to significantly reduce infections. The influence of these various strategies relative to other non-pharmaceutical interventions remains to be investigated. It has been suggested^16^ that with more data, implicit Randomized Controlled Trials may be carried out by comparing similar localities enforcing different subsets of policies.

The difference in results for data-driven mobility and those from traditional homogeneous models (Supplementary Fig. S7) can be attributed to information-structural properties of human mobility, creating a complex *network effect*. The inclusion of social and economic considerations are likely to add to these complexities, for example, it has been argued that the worldwide food supply chain is being affected by labor shortages, trade restrictions, and factory closures^24^. The relations between mobility, essential industries, and economics will be important to decipher for the development of long term strategies.

In conclusion, from the perspective of actions to control the pandemic, our results suggest interventions on highly mobile people and most popular venues are most likely to be effective. These interventions can take forms of priority in testing, vaccination, protective equipment, or stricter regulations. The infection spread at venues can take the form of limiting large gatherings, reducing the need to visit the venue or different modes of operations (e.g. deliveries and takeaways)—and should be encouraged more at popular venues. Our model and simulation methodology can be used to identify the points of action in other datasets and environments.

We found that large scale general lockdowns may not reduce the total number of infections in all cases, but they can be used to delay infections and lower the peak. Thus, it can be used to temporarily avoid overloading hospitals, and to gain time to build up resources. Partitioning a population into cohorts can be useful in small populations (such as small schools). In large groups where they are harder to implement, they are also less likely to effective if implemented.

We note that our analysis is purely on the mobility dimension. The spread of the infection depends on various other factors, such as the nature and architecture of the venue (indoor vs. outdoor), awareness of people, and other complex behavioral factors that should ideally be taken into account. Mobility patterns themselves are dependent on economic and industrial aspects that are beyond the scope of our study. More research is needed for detailed models incorporating complex aspects of infection spread.

## Method

Our work takes the approach of multi-agent models but incorporates real mobility traces to study the probable spread of an infectious disease. The study involves three data sets that represent three typical social settings. (Refer to Supplementary material for detailed data description).

- *Foursquare check-ins.* We use check-in data^25,26^ from the Foursquare mobile application, which captures snapshots of human mobility in popular public spaces. The dataset includes seven major cities across the world (New York, Chicago, Los Angeles, London, Tokyo, Istanbul, and Jakarta), and contains a total of 2,293,716 check-ins from 24,068 individuals in 397,610 venues over a period of 140 days.
- *University campus*^27^: WiFi log data on connections of mobile devices with nearby WiFi access points from a large university campus (Tsinghua University) is a representative case of mobility in a large university setting. The dataset contains 106,975 average daily localization points from 47,359 individuals.
- *Personal electric bikes*^28^: Densely sampled GPS mobility traces of 46,078 personally owned electric bikes over the period of a month provides a unique dataset as they capture unstructured personal mobility patterns not tied to specific public spaces or institutions.

All three data sets have fine-grained location resolution of within 10s of meters, which allow us to investigate inter-personal interactions and interactions of individuals and public venues. These datasets collectively cover three representative slices of human mobility that play important roles in the spread of infectious diseases. Infection can propagate person to person at a meeting event (when two agents are in proximity), or through a venue (when a susceptible agent visits a venue recently visited by an infectious agent). Note that these datasets were collected pre-COVID between 2012 and 2018, representing “normal” human movements. We ran simulations on the three datasets with various intervention strategies and show their impact in slowing down the spread. We study the total number of individuals infected under various intervention strategies, the growth rate of new infections, the maximum number of infected individuals at any time (height of the peak), and the time-varying reproduction number ($R_{t}$). The social cost of an intervention strategy is measured as the number of check-ins and meetings lost due to the strategy.

### Mobility data driven infection simulation model for COVID-19

We customize the SEIR model^8^ to operate on mobility data and incorporate the properties of COVID-19 transmission as probabilistic features using the parameters from Center for Disease Control and Prevention^29,30^ (Fig. 1 Part I). Like the SEIR model an agent in our model can be in one of the four states: susceptible, infected (or exposed), infectious, and recovered or immune. Unlike the standard model that assumes person-to-person disease transmission through uniformly probable contacts, we obtain person to person contacts from the mobility datasets and extend the model to disease transmission via check-ins to venues.

The simulation starts by infecting a random set of *seed agents* with all other agents as susceptible. The disease transmits from person to person at a *meeting* (in the University and the Bike dataset), or through a venue (in the Foursquare datasets) with a transmission probability, $\beta$. In the University dataset, two agents meet when they are at the same location (such as a classroom or cafeteria) at the same time. A meeting occurs when two bikes are within 5 m for more than 5 min in the Bike dataset. When an infectious agent visits a venue in the Foursquare dataset, we mark the venue as infected and it remains infected for 48 h. This aligns with the studies on COVID-19 lifespan on various surfaces^31^.

A susceptible person is infected with probability $\beta$ when meeting an infectious person or visiting an infected venue. The value of $\beta$ is determined for each scenario based on knowledge of existing variables such as $R_{0}$ (see Supplementary material for details). An infected person remains in incubation for $\mathcal {N}(6, 1)$ days followed by the infectious stage gaining the ability to infect other susceptible agents or venues. Here, $\mathcal {N}(\mu ,\sigma )$ denotes a sample from a normal distribution with mean $\mu$ and standard deviation $\sigma$. The distribution models variability in infection behavior. For COVID-19 there are a significant fraction of asymptomatic carriers^32,33^. As CDC mentioned, we consider $35\%$ of the infected population (randomly chosen) to be asymptomatic. A symptomatic person remains infectious for $\mathcal {N}(5, 1)$ days before showing symptoms and subsequently enters the *in-care* stage such as at home self-quarantine or hospitalization. At this stage, the person ceases to infect others. After $\mathcal {N}(13, 1)$ days in care, the agent assumed to recover and become immune. On the other hand, asymptomatic agents do not seek care, and thus they skip the in-care state and remain infectious for $\mathcal {N}(18, 1)$ days.

We study temporal dynamics of the infection spread in four dimensions—the total number of infected people till date, number of active cases, number of new infections, and epidemiological parameters such as the time-varying reproduction number ($R_{t}$) or growth rate ($\lambda _{t}$). The active cases include individuals that have been infected till date but not yet recovered. The time-varying reproduction number^34^, $R_{t}$ for day *t* is the expected number of average individuals infected by a single agent who gets infected on day *t*. The growth rate, $\lambda _{t}$, is the ratio of the total number of agents newly infected on the day *t* to the same number on the day $t-1$.

### Ethics of data and methodology

In this study, we used secondary datasets collected in the past (between 2012 and 2018) by other researchers, who have published detailed information about the corpora and the collection processes^25–27^. The project has gone through the ethics review process at the School of Informatics, University of Edinburgh, and has received a waiver for use of these secondary corpora. More specifically, the datasets were completely anonymous with personal identifying information removed. They are either public or collected with consent as follows:

- The Foursquare data were derived from open public posts on Twitter between 2012 and 2014 by researchers at University of Freibiurg and made available online (https://sites.google.com/site/yangdingqi/home/foursquare-dataset). Details of the data has been published previously^25,26^.
- The University and Bike datasets were collected from adult participants with informed consent. The University dataset was collected between September 2015 to November 2015 by the research group at (NetMan) AIOps labs at Tsinghua University, for research purposes. The data collection process was published in 2016^27^. We received the dataset through personal communication with the authors. The Bike dataset was collected between June 2018 and September 2018 by a private company, Zhejiang Tendency Technology Co., Ltd in the city of ZhengZhou, P.R. China.

## Supplementary Information

Supplementary Information.

## Data Availability

Among the data used in the work, the Foursquare check-ins dataset spanning seven cities are publicly available and has been cited in the text. The Tsinghua WiFi dataset and Zhengzhou bike datasets cannot be made public due to our non-disclosure agreement with the respective data owners. Our contribution is the program code for the simulations and analysis, which is publicly available at https://github.com/SBUhaotian/Mobility_Contagion^35^ for reproducibility, and use on other datasets.
