# Supplementary material for: Heterogeneous interventions reduce the spread of COVID-19 in simulations on real mobility data
Source: Sci Rep. 2021 Apr 8;11:7809. doi: 10.1038/s41598-021-87034-z (PMC8034422; doi:10.1038/s41598-021-87034-z)
Supplement: Supplementary file 1 — Supplementary Information. [file 41598_2021_87034_MOESM1_ESM.pdf]

Heterogeneous Interventions Reduce the Spread of COVID-19 in Simulations on Real Mobility Data

Haotian Wang[1,+], Abhirup Ghosh[2,+], Jiaxin Ding[3], Rik Sarkar[4,*], Jie Gao[1]

[1] Department of Computer Science, Rutgers University, USA
[2] Department of Computer Science and Technology, University of Cambridge, UK
[3] John Hopcroft Center for Computer Science, Shanghai Jiao Tong University, China
[4] School of Informatics, University of Edinburgh, UK

[*] To whom correspondence should be addressed; E-mail: rsarkar@inf.ed.ac.uk.
[+] these authors contributed equally to this work

SUPPLEMENTARY MATERIAL

Data

All our datasets describe individual mobility by timestamped locations. Datasets use anonymized individual identifiers and have assorted temporal and spatial resolutions. Table S1 quantifies the datasets.

Foursquare dataset. Foursquare enables users to record their check-in times at venues and thus produce a log of users' mobility to public places. Although the check-ins are accessible to people only within social circles, many people choose to share them on Twitter. Authors in[1,2] collected the Foursquare check-ins posted on Twitter. In this work, we use a sub-sample of the available dataset containing 7 cities (New York, Tokyo, Istanbul, Chicago, Jakarta, Los Angeles, and London) with dense data distributions spanning between Apr-2012 and Aug-2012. Check-ins for a city are selected by first choosing a Geo-location bounding box containing the city, for example, we consider the venues within the box ranging between latitude $[40.5378°N, 41.0131°N]$ and longitude $[73.4532°W, 74.3074°W]$ are considered in the New York City dataset. Then we select the check-ins that happened in the selected venues. Essential characterizations of the datasets agree with the findings in[1,2]. The check-ins have the temporal resolution in seconds.

University dataset. The Tsinghua University campus covers an area of $4.4km^2$ with $47,359$ students and $12,000$ faculties. There are $2,346$ wireless access points deployed in 114 buildings including classrooms, departments, administrative buildings, apartments, gyms, libraries, restaurants, supermarkets, and hotels. The dense deployment allows tracking the connected devices. Each connected device reports its connecting access point every 5 minutes. The dataset[3] is one week long and assuming that the weekly movement stays similar in this setting, we repeat the dataset to generate more days. When two agents stay in the same room within a period, it is regarded as a meeting between them.

Bike dataset. Zhengzhou (China) has wireless tracking terminals that can locate and monitor electric bikes in real-time using a variety of IoT sensors, such as satellite positioning module, voltage and current detection module, and temperature sensing module. Our dataset[4] has timestamped GPS locations of $46,087$ personally owned electrical bikes with the temporal granularity of 10 seconds. On average, there are more than $41,000$ people traveling by electrical bikes and more than 19 million data points collected every day for a month and repeat in the simulation. If a person stops moving in one location for more than 5 minutes, this location with the period is regarded as a stay point of this agent. On average, there are $118,337$ daily stay points and $90,144$ meetings every day.

Data resolution. The data we operate with has a high spatial resolution, which allows us to execute detailed agent-based models. Other forms of data often used in movement analysis include cellular data - representing cell tower connections of mobile phones, including errors at a scale of kilometers, which is the range of cell towers. Even in the urban scenarios of dense deployments, cell towers are separated by hundreds of meters implying correspondingly low resolutions. In comparison, in the bike GPS dataset, we have errors at the level of few meters, and similarly, the errors in the University WiFi dataset are bounded by a few tens of meters, which is the typical WiFi range, and connection to the same WiFi usually implies agents being in the same building. The Foursquare dataset correspondingly contains precise information of agents visiting the same venue. Compared to metapopulation models that treat the population as a set of communities, this high-resolution simulation incorporates a more dynamic and precise movement pattern. We are able to simulate movement or presence of agents with precision such as presence in the same building, and correspondingly likely infection transmission.

Intervention Strategies

We have investigated multiple non-pharmaceutical interventions. Some strategies referenced briefly in the main article are described below.

Intervening random individuals and venues. Here, the venues to close (Supplementary Fig. S11 online) and people to protect (Supplementary Fig. S12 online) are chosen uniformly at random. In the Foursquare, NYC dataset, closing 40% random venues results in $\sim 20\%$ reduction in total infected people and 60% reduction in the social value. In other Foursquare datasets of cities such as Istanbul and Tokyo, the effect of this strategy on health value is minimal with a reduction of not more than $\sim 5\%$. Other datasets show a similar trend. In the University dataset, closing 40 random venues brings 17% reduction in total infected people. Across all datasets, closing 40% random venues results in more than $\sim 33\%$ reduction in the infected population (Supplementary Fig. S12 online).

These strategies are shown to be less effective than intervening the most active agents or most popular venues. In the Foursquare NYC dataset, to achieve the same social value ($\sim 60\%$), the strategy to close randomly chosen venues achieves 65% less health value compared to the targeted strategy to close the most popular venues. In the University dataset, to achieve the same social value ($\sim 80\%$), the strategy to protect random people obtains 55% less health value compared to the strategy to protect the most active people.

Methods: Implementation and Experimental setup

The Foursquare datasets contain time-ordered check-ins along with categorical unique identifiers for venues and agents. In the University and Bike datasets, meetings are computed and stored as data pre-processing steps. The meetings in the University dataset are computed first grouping the contacts by venues and then finding the overlapping staying intervals. In the Bike dataset, the spatial proximity is computed by a kd-tree, a spatial indexing structure to find the location within a given distance. The meetings are stored with the participates, location, starting, and ending time. Meetings are ordered by their starting time.

The simulation progresses sequentially over the events ordered by check-ins or meetings and keeps track of the states of the agents and venues. While the state machine for the agents is described in the main article, a venue can be in one of the two states – susceptible and infected. Initially, all venues are susceptible and a venue becomes infected once an infectious agent checks-in. Each infected venue keeps a timer that goes off after 48 hours of being infected and then the venue becomes susceptible again. If an infectious agent visits an infected venue, the timer is reset to the current check-in time. We maintain the timer by operating on it only at check-in event times.

In the person to person infection spread model, the infection probability, β, used in the simulation is derived for a particular dataset based on existing knowledge of the initial reproduction number, R_0 for COVID-19. R_0 denotes the expected number of people infected from a single person, i.e., $R_0 = c\beta T$, where c is the average number of daily meetings per person, and T is the average number of days an agent remains infectious. While c is estimated from a dataset, model parameters in Fig. 1B give the value of T as 9.55 days. For the disease transmission through venues, β has a different interpretation and does not correspond to usual parameters such as R_0. We evaluate a range of β value in Supplementary Fig. S14 and find consistent patterns across them.

There are 10 initial infectious seeds at the beginning of each simulation. The infection probability is set as 0.75 for the Foursquare dataset and takes R_0 as 3 to obtain the University and Bike dataset's infection probabilities.

For both the person to person and via venue transmission models, the probabilistic virus transmission is simulated as follows. A uniform random number is sampled in the range $[0,1]$ and compared against the infection probability, β. The probabilistic experiment is successful, i.e., the agent becomes infected, if the sample is less than β.

We study dynamics of the infection spread in four dimensions – the total number of infected people till date, number of active cases, number of new infections, and epidemiological parameters such as the time-varying reproduction number (R_t) or growth rate (λ_t). The active cases include individuals that have been infected till date but not yet recovered. The time-varying reproduction number, R_t for the day t is the expected number of average individuals infected by a single agent who gets infected on the day t. The growth rate, λ_t, is the ratio of the total number of agents newly infected on the day t to the same number on the day $t-1$. When λ_1 above 1, the number of infections grows exponentially; when λ_t is below 1, the number of new infections converges.

In the contact graph-based study, the contagion simulation uses the same model and parameters as mobility datasets (Fig. 1 Part I). It starts with 10 initial randomly selected infectious nodes (*seeds*) and all other nodes are at the susceptible state. It works in synchronous and discrete-time rounds – at each round, an infectious node infects its susceptible neighbors in G with a probability, p. As both the meetings and the check-ins to venues are abstracted to counts instead of timestamped events, the contacts are considered to be distributed uniformly over the duration of the dataset, and infection transmission probabilities β are adjusted per edge as $p = (1-(1-\beta)^w)$. In the infection transmission model via venue, an infectious individual infects a venue with probability $\min(1,w)$ at each round, and the infected venue in-turn infects an individual with probability p.

Similar to mobility-based simulations, in the social network setting we use $\beta = 0.75$ for transmission via venues. In the person-to-person transmission model, β is derived from R_0 from the same expression, $R_0 = c\beta T$. Here, c is counted as the average total weight from a node and T remains the same as 9.55.

Computation of social and health values of interventions. The social value of an intervention strategy is measured as the percentage of activities (check-ins) preserved under the intervention. When a venue is closed or an agent is protected, the corresponding activities are canceled. The social value of a strategy is denoted by $1 - x/y$ in percentage, where x and y number of check-ins (for Foursquare datasets) or meetings (for University and Bike datasets) performed under the intervention and without intervention respectively. The health value of an intervention is correspondingly measured as the percentage of agents who escape infection due to the intervention. If x and y number of agents are infected under intervention and without intervention respectively, then the strategy's health value is the proportion $1 - x/y$, usually written as the percentage.

The simulations are implemented in Python and executed in standard desktop machines with Intel i7 cores and 16GB memory. The simulation is compute-efficient – all our simulations run under 5 minutes. The efficiency results from in-memory processing, pre-computation of meetings prior to simulation, and storing infection states in the data structures for venues. All of these reduce the number of operations at each discrete event timestamp. The result of a simulation contains the timestamps of new infections and recoveries.

As our simulation model is stochastic, each experiment is run 10 times to test their stability. We report the temporal dynamics in the figures with the median of 10 runs as the solid curve and the shaded regions denote the area between 25 and 75 percentiles. The figures are smoothed with 7 day rolling average. Moreover, to bring about the major trends, the growth rate and reproduction number plots are further smoothed using a standard Gaussian filter with the standard deviation of the Gaussian kernel set as 2 days.

Existing Models for infection spread

There are various existing models for infection spread. We discuss below the ones most relevant to us.

- *Epidemiology models:* Classical models in epidemiology include SIR (Susceptible-Infected-Recovered), SEIR (Susceptible-Exposed-Infected-Recovered) models[5] and other variations[6,7]. These models make simplistic assumptions of a homogeneous population and any two individuals have the same structure of interactions and dynamics. The model evolves by using a small number of parameters such as an infectious person's chance infecting another one and then deriving ordinary differential equations. To incorporate heterogeneity in a large population, meta-population models[8] include population structures that describe variations in age groups, behaviors, neighborhoods, but in general, these models are coarse-grained.

- *Data-driven models:* A few models such as the one from the Institute of Health Metrics (IHME)[9] use a data-driven model to predict the number of new infections, based on data from other countries. This model assumes that the infection process is uniform across different countries, thus it ignores the important parameters such as the discrepancy of culture, weather, the density of population, and the lifestyle.

- *Multi-agent models:* The models from Imperial College London[10] are individual-based multi-agent models. These models are fairly complicated with a large number of parameters describing the interactions between the agents. For example, individuals are assumed to reside in high-density residential areas from census data. Contacts with other individuals are assumed to happen within the household, at school, in the workplace, and in the wider community. The parameters of population density in these scenarios are taken as the average in published data. It is a challenge to choose these parameters and validate the choices against real data.

Comparison with standard SEIR model

From a dataset, we count the average number of daily contacts for an agent, c. With the population size of N, there are $Nc/2$ contacts in total per day. The SEIR simulation progresses in synchronous daily rounds and $Nc/2$ contacts are randomly sampled each day. The other parameters remain similar to Fig. 1.

We apply the standard SEIR model to COVID-19 parameters, i.e., consider meetings between random pairs of agents. We simulate the person-to-person transmission model in the University and the Bike datasets keeping the model parameters the same as Fig. 1B. Given the dataset, $Nc/2$ contacts between two agents are randomly sampled. Here, we ignore the time-stamped of each meeting from the dataset. Similar to our simulation model, the simulation starts with 10 initial seeds and proceeds with probabilistic disease transmission using sampled contacts.

Supplementary Fig. S7 compares the infection spreads in two models for a setting without intervention. For both University and Bike datasets, a larger population gets infected by the SEIR model compared to our mobility based simulation. In both the datasets, the peak of active infections in the SEIR model is at least 15% higher delayed by more than 35 days than the mobility model. This is due to heterogeneity of agents – more active agents get the virus early and infect other susceptible agents early – resulting in an early peak. Besides a large fraction of agents have a low number of meetings, therefore, have less

risk of being infected which leads to a lower total infection number compared with the SEIR model. Our observations match with the observations in[11].

The difference in meeting distributions in the two simulation models results in different distributions for the number of agents infected from an individual. While the mobility-based model has a long tail distribution suggesting that the more active agents infect more people, the SEIR model does not have a long tail.

References

1. Yang, D., Qu, B., Yang, J. & Cudre-Mauroux, P. Revisiting user mobility and social relationships in lbsns: a hypergraph embedding approach. In *The World Wide Web Conference*, 2147–2157, DOI: https://doi.org/10.1145/3308558.3313635 (2019).
2. Yang, D., Qu, B., Yang, J. & Cudre-Mauroux, P. Lbsn2vec++: Heterogeneous hypergraph embedding for location-based social networks. *IEEE Transactions on Knowl. Data Eng.* DOI: https://doi.org/10.1109/TKDE.2020.2997869 (2020).
3. Sui, K. *et al.* Characterizing and improving wifi latency in large-scale operational networks. In *Proceedings of the 14th Annual International Conference on Mobile Systems, Applications, and Services*, 347–360, DOI: https://doi.org/10.1145/2906388.2906393 (2016).
4. Wang, H. & Gao, J. Distributed human trajectory sensing and partial similarity queries. In *2020 19th ACM/IEEE International Conference on Information Processing in Sensor Networks (IPSN)*, 253–264, DOI: https://doi.org/10.1109/IPSN48710.2020.00-43 (IEEE, 2020).
5. Wang, H. *et al.* Phase-adjusted estimation of the number of coronavirus disease 2019 cases in wuhan, china. *Cell discovery* **6**, 1–8, DOI: https://doi.org/10.1038/s41421-020-0148-0 (2020).
6. Lai, S. *et al.* Effect of non-pharmaceutical interventions to contain covid-19 in china. *Nature* DOI: https://doi.org/10.1038/s41586-020-2293-x (2020).
7. Prem, K. *et al.* The effect of control strategies to reduce social mixing on outcomes of the covid-19 epidemic in wuhan, china: a modelling study. *The Lancet Public Heal.* DOI: https://doi.org/10.1016/S2468-2667(20)30073-6 (2020).
8. Chinazzi, M. *et al.* The effect of travel restrictions on the spread of the 2019 novel coronavirus (COVID-19) outbreak. *Science* **368**, 395–400, DOI: https://doi.org/10.1126/science.aba9757 (2020).
9. IHME COVID health service utilization forecasting team, M. C. Forecasting covid-19 impact on hospital bed-days, icu-days, ventilator-days and deaths by us state in the next. *MedRxiv* **4**, DOI: https://doi.org/10.1101/2020.03.27.20043752 (2019).
10. Ferguson, N. M. *et al.* Imperial college COVID-19 response team. impact of non-pharmaceutical interventions (NPIs) to reduce COVID-19 mortality and healthcare demand. published march 16, 2020, DOI: https://doi.org/10.25561/77482 (2020).
11. Stehlé, J. *et al.* Simulation of an seir infectious disease model on the dynamic contact network of conference attendees. *BMC medicine* **9**, 87, DOI: https://doi.org/10.1186/1741-7015-9-87 (2011).

Table S1: The statistical characteristics for the Foursquare dataset.

	Number of check-ins	Number of people	Number of venues
New York	202,599	1,790	39,764
Istanbul	559,966	8,925	53,075
Tokyo	642,687	4,744	96,931
Chicago	103,432	924	23,391
Los Angeles	402,989	3,590	104,629
Jakarta	336,386	3,623	67,047
London	45,657	472	12,773

Table S2: The statistical characteristics for University dataset and Bike dataset.

	# of daily stay points	# of people	# of venues	# of daily meetings
University	106,975	47,359	2,346	2,476,837
Bike	118,337	46,087	N.A.	90,144

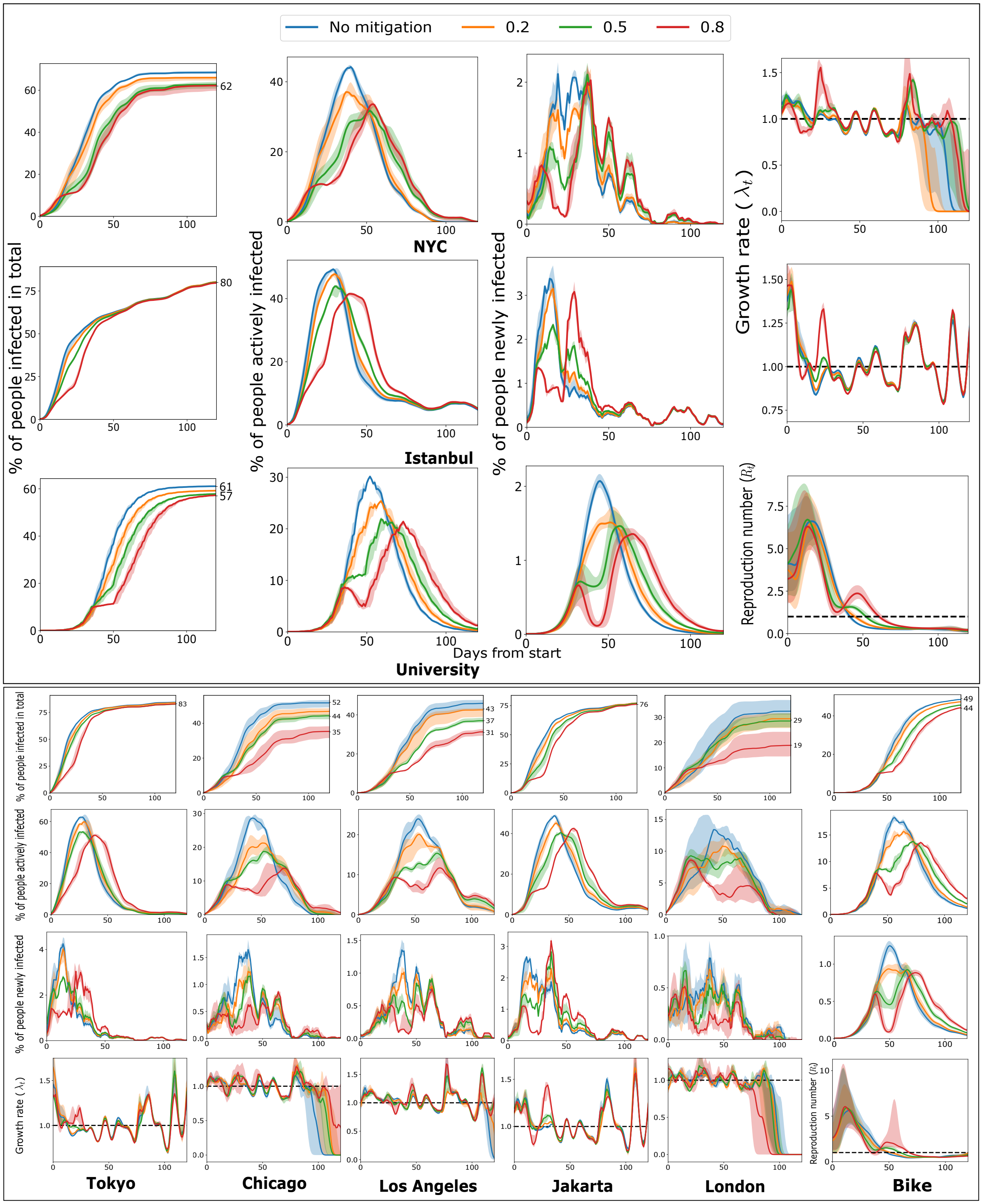

Figure S1. Infection spreading with the intervention strategy of varying probability, with which check-in is skipped with random sampling. All interventions start when 10% of the population is infected and it lasts for 15 days. While with higher probability (i.e., stronger intervention) the peak of active infections gets delayed and lowered, the total number of infected people is independent of the probability (in Istanbul, Tokyo, Jakarta, University, and Bike datasets.

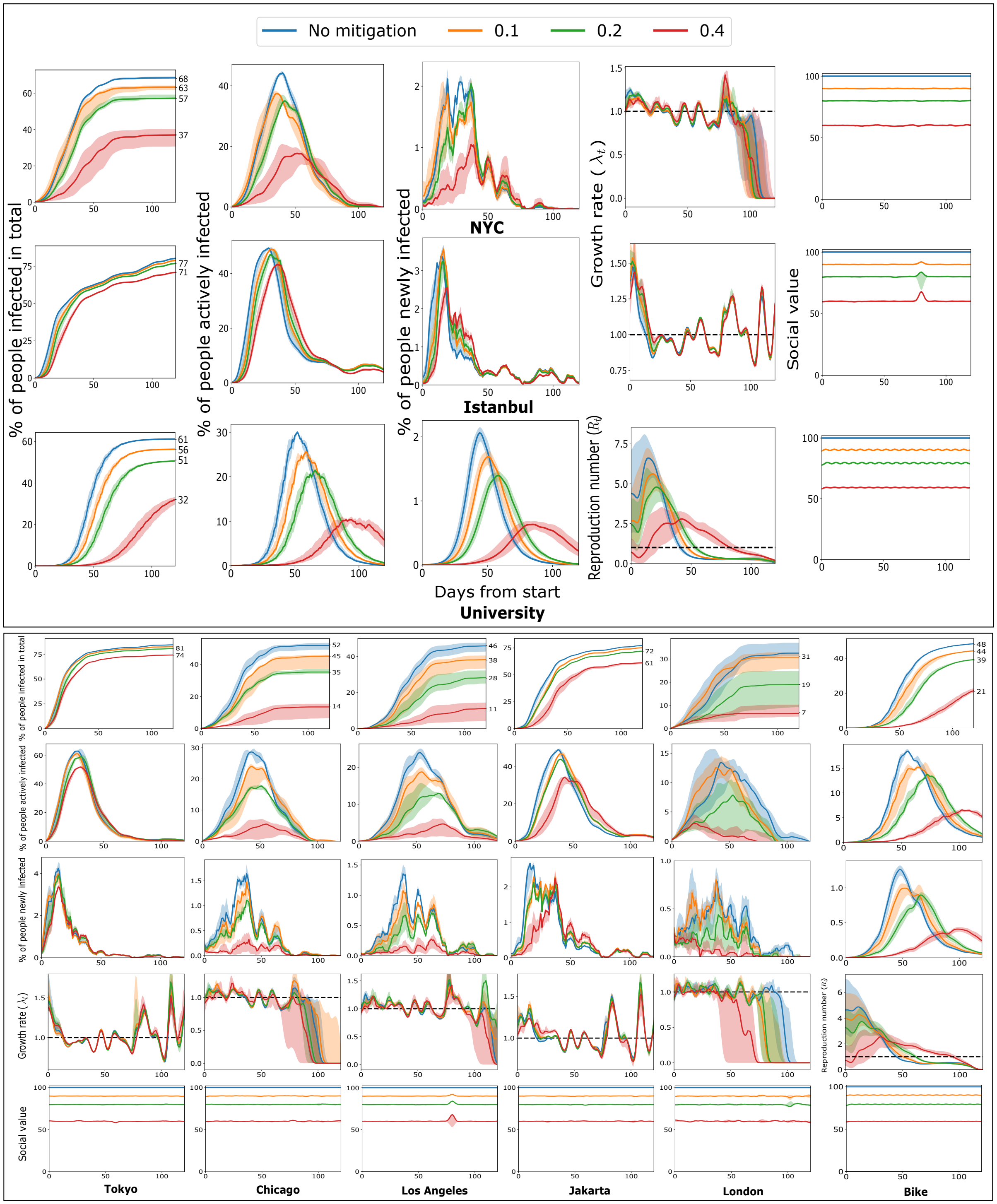

Figure S2. Infection spreading with the intervention strategy of Stay home intervention. This intervention strategy uniformly randomly ignores a check-in with a probability. This represents people reducing their social activities. Naturally, as more check-ins are skipped, the infection is slowed, and the social value is also reduced.

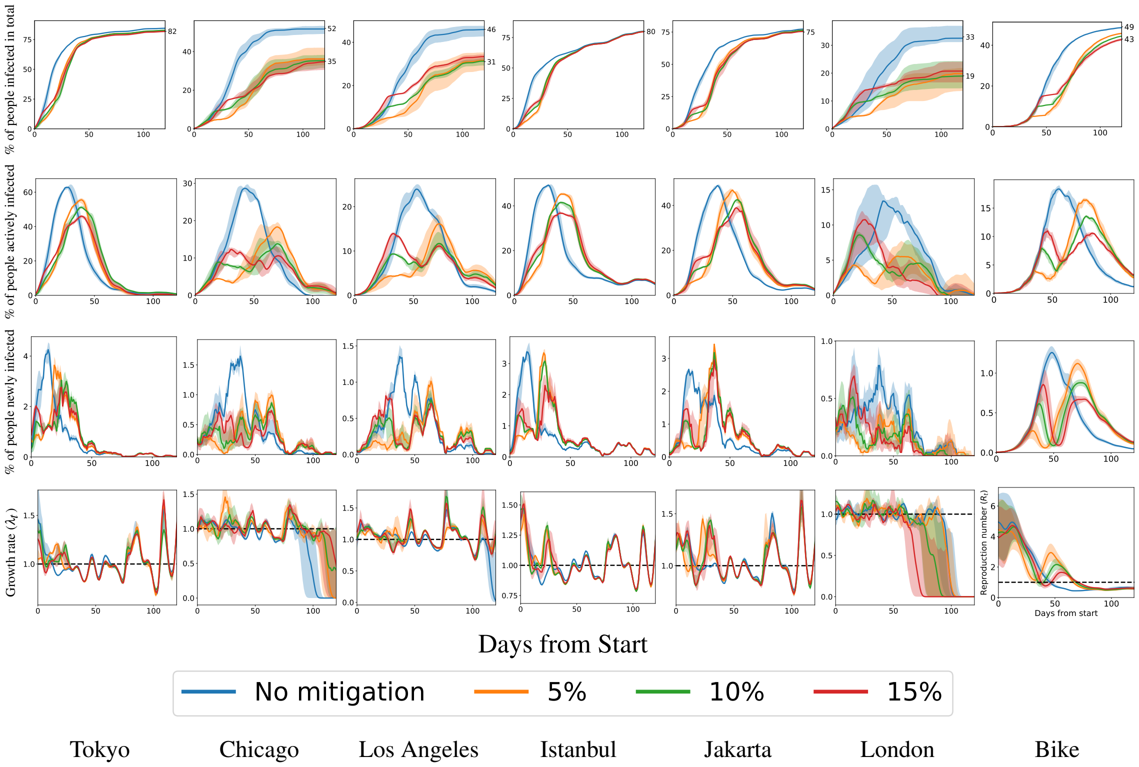

Figure S3. Infection spreading with a varied start time of uniform intervention as Part I in Figure 2. The intervention strategy uniformly randomly skips 80% of the check-in or meeting events. Intervention starts when 5%, 10%, and 15% population is infected and lasts for 15 days. This intervention strategy can reduce the total number of infected agents in some datasets, but the fraction is independent of the starting time of the intervention.

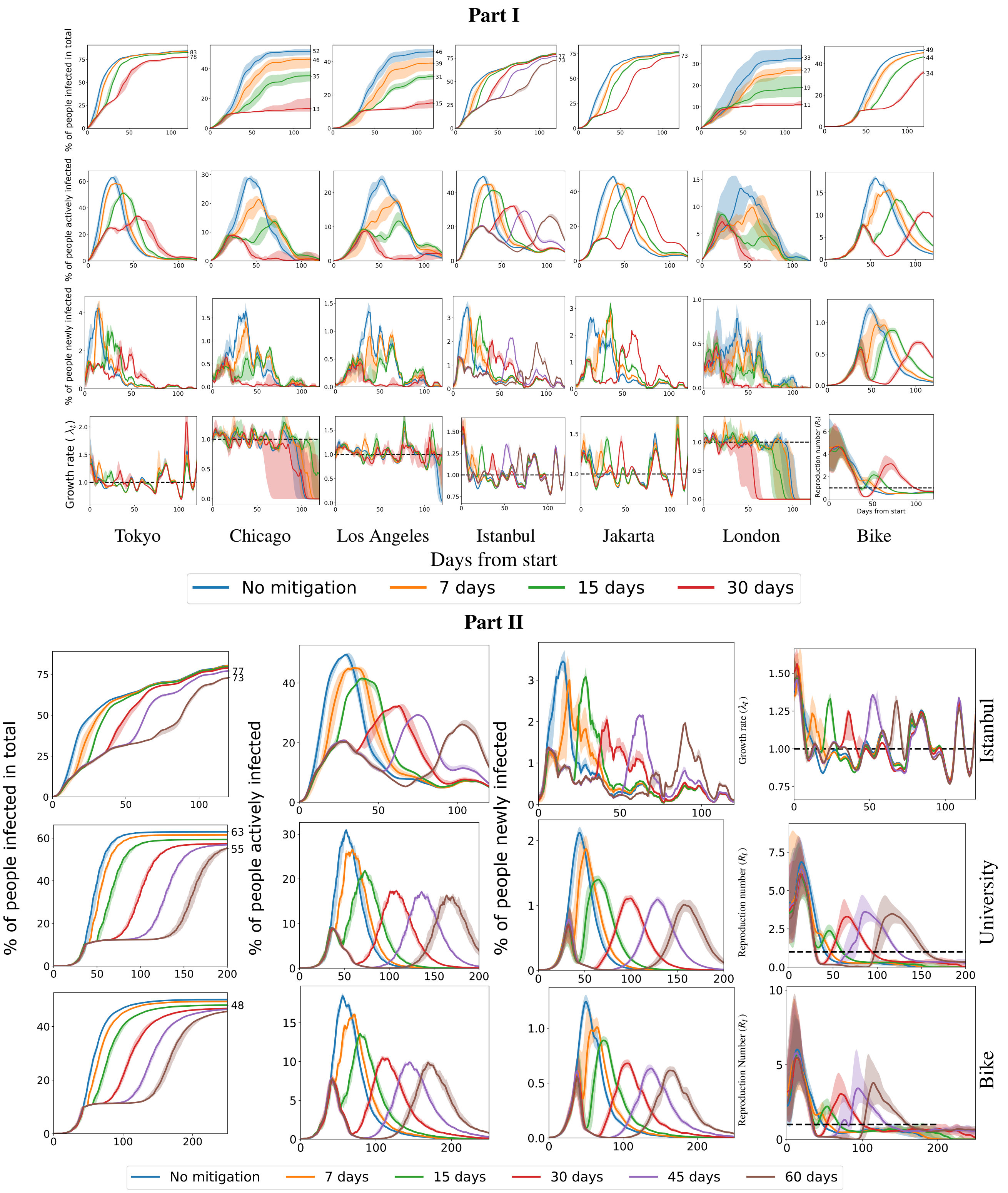

Figure S4. Infection spreading with the intervention strategy of varying intervention length similar to Part II in Figure 2. With long intervention, the total number of infected people is reduced significantly in Chicago, Los Angeles, London, and Bike datasets. Here, 30 days are enough to have the infected people recovered, and the second wave of infection does not occur. In the other datasets, after the intervention, there is a second wave of infection, leaving a similar number of infected people. We also test longer interventions in Part II in the datasets with larger populations and the patterns in Part I hold.

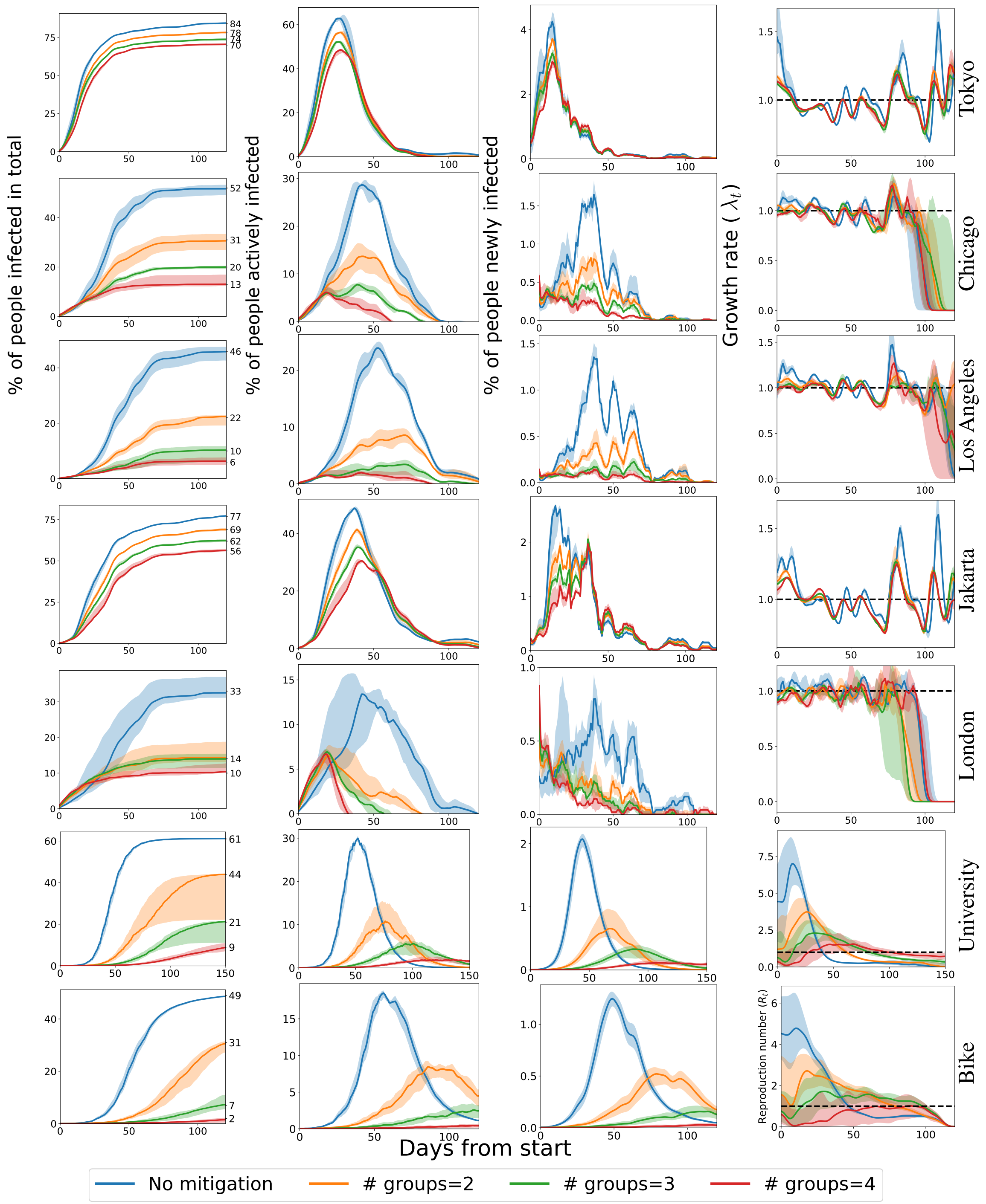

Figure S5. Infection spreading with the intervention strategy of dividing people into groups. With more groups, each group has fewer agents, and the connections between them become sparser. In most datasets, dividing people into 4 groups can reduce the total number of infected people significantly.

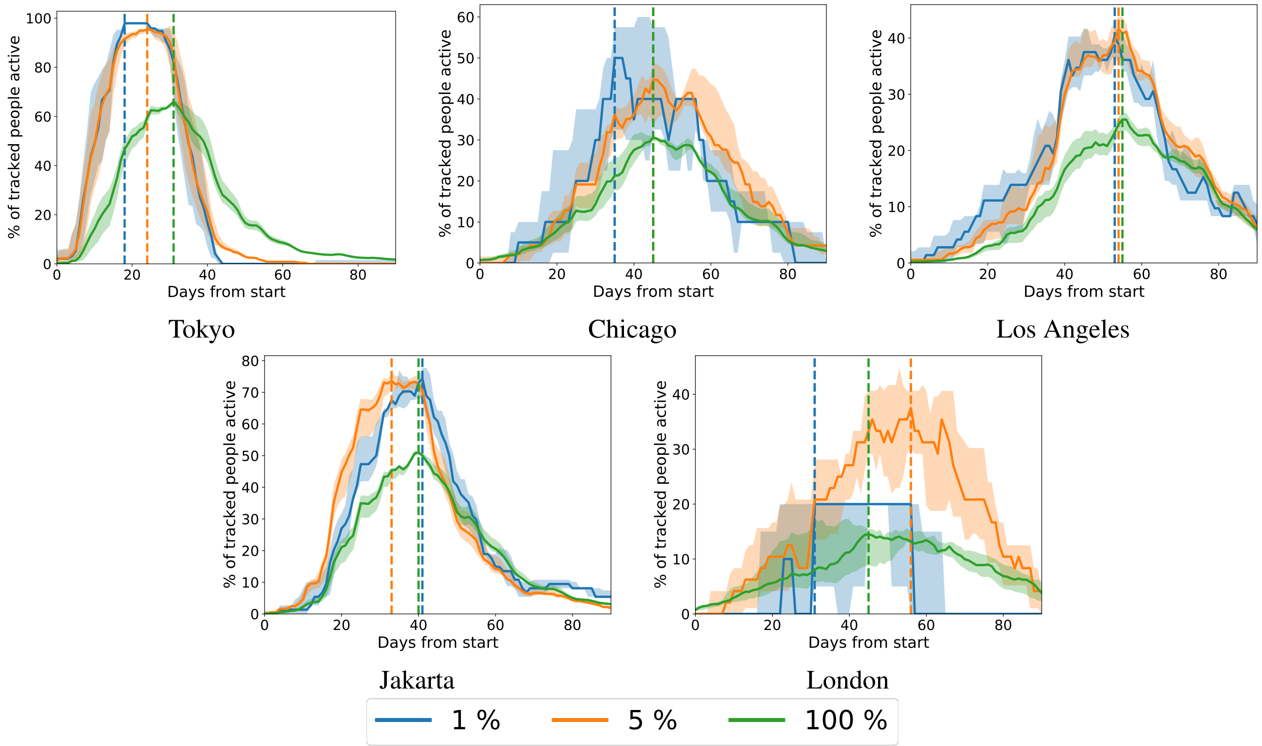

Figure S6. Tracking the more active agents similar to Figure 3. The vertical lines denote the days of the peak. All the datasets show consistent trends that the most active agents get infected with a higher proportion and earlier than the whole population.

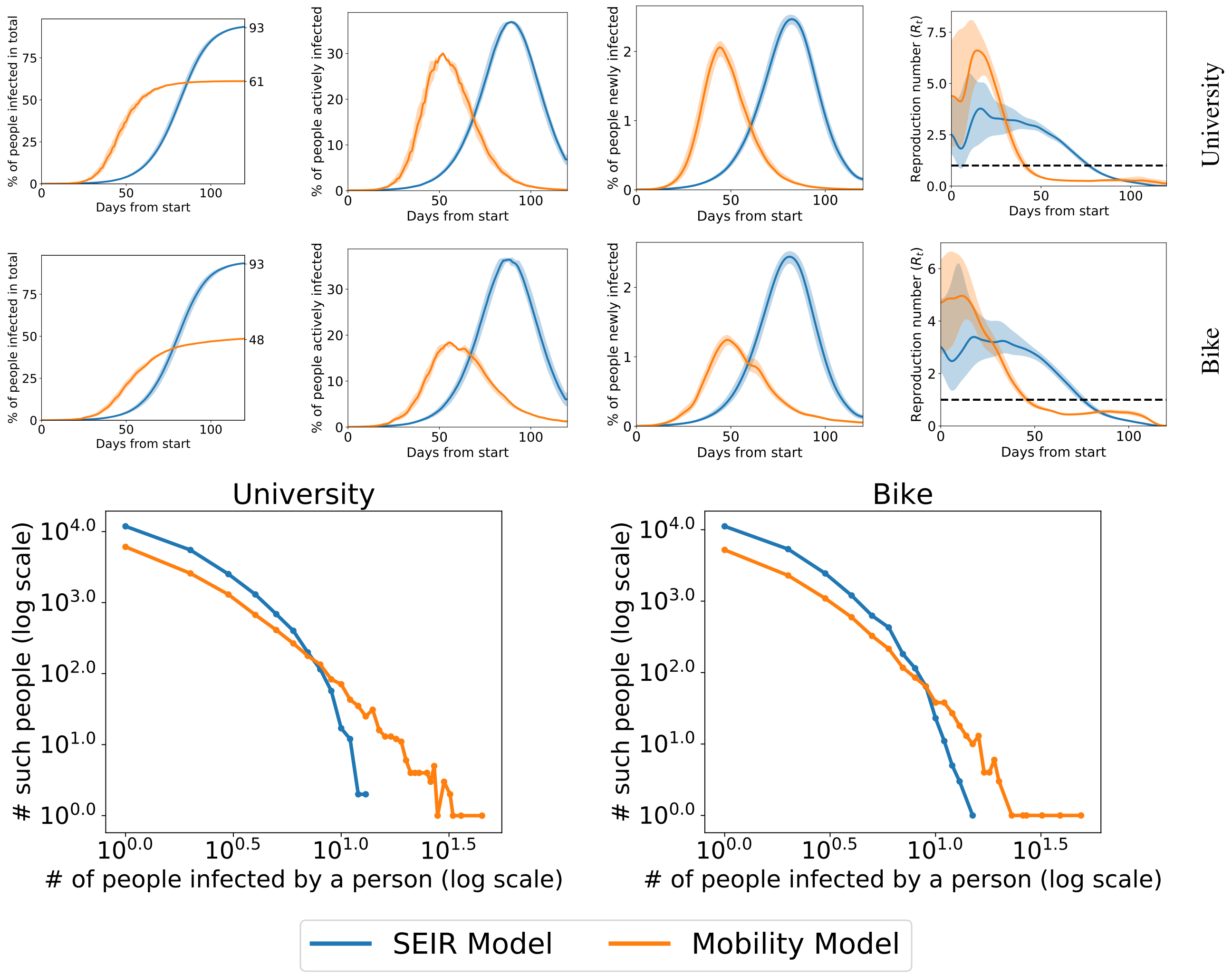

Figure S7. Infection spreading with random meeting information and real mobility data. In the SEIR model, almost all people get infected in the end, which is a much higher percentage than in the mobility model. At the same time, the peaks of people actively infected in the SEIR model are about 35 days delayed, compared with the mobility model in two datasets. The third row shows the distribution of the number of people infected by a person. In the mobility model, an agent can infect at most 50 susceptible agents, while in the SEIR model, at most only 15 susceptible agents can be infected from one infectious agent. Thus, there is a long tail in the mobility model because there are some more active agents, who spread the disease quickly.

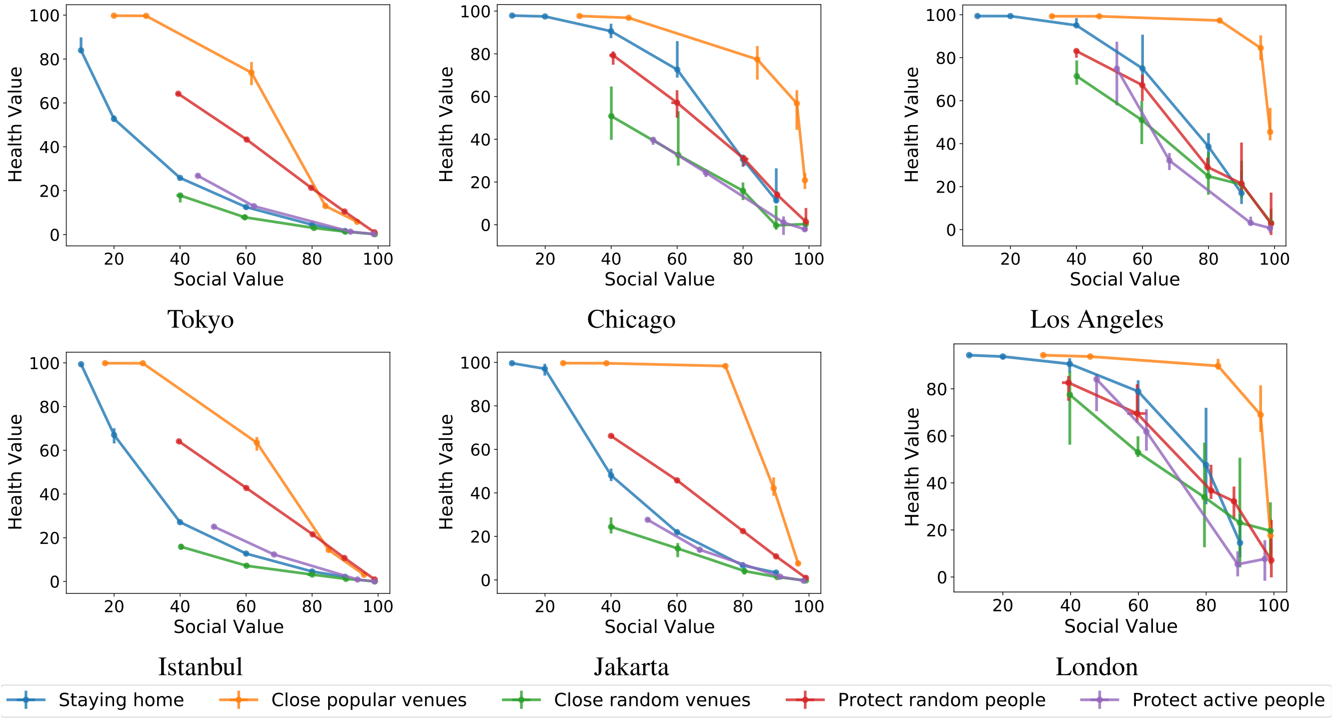

Figure S8. Comparison of different intervention strategies in all other Foursquare datasets similar to Figure 4. Closing popular venues is the most advantageous from both health and social value perspectives.

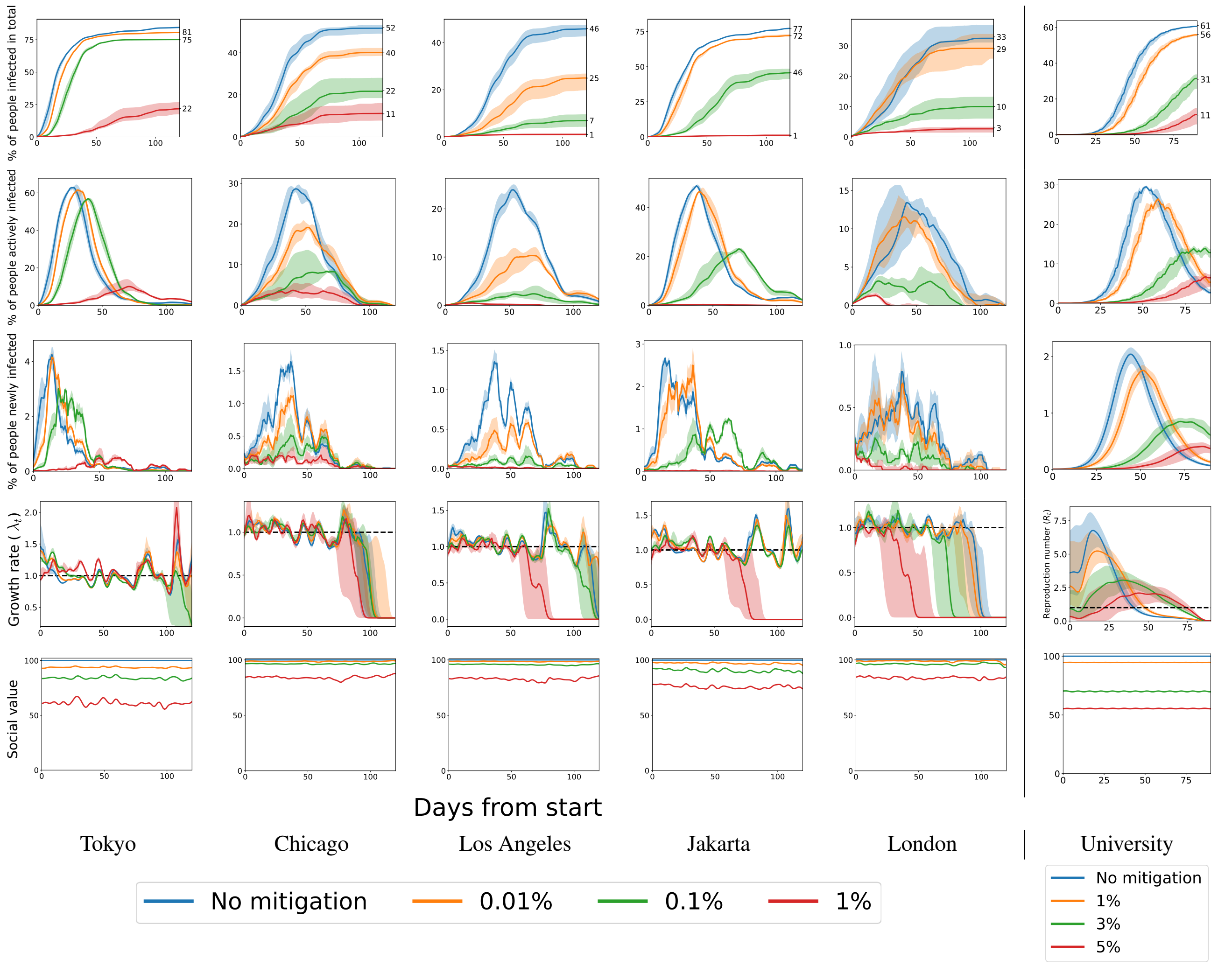

Figure S9. Infection spreading with the intervention strategy of closing popular venues. With more popular venues closed, the total number of infected people is reduced. The peaks of people actively infected are delayed and become lower. In addition, closing these popular venues has a comparatively smaller influence on the social value than uniform intervention.

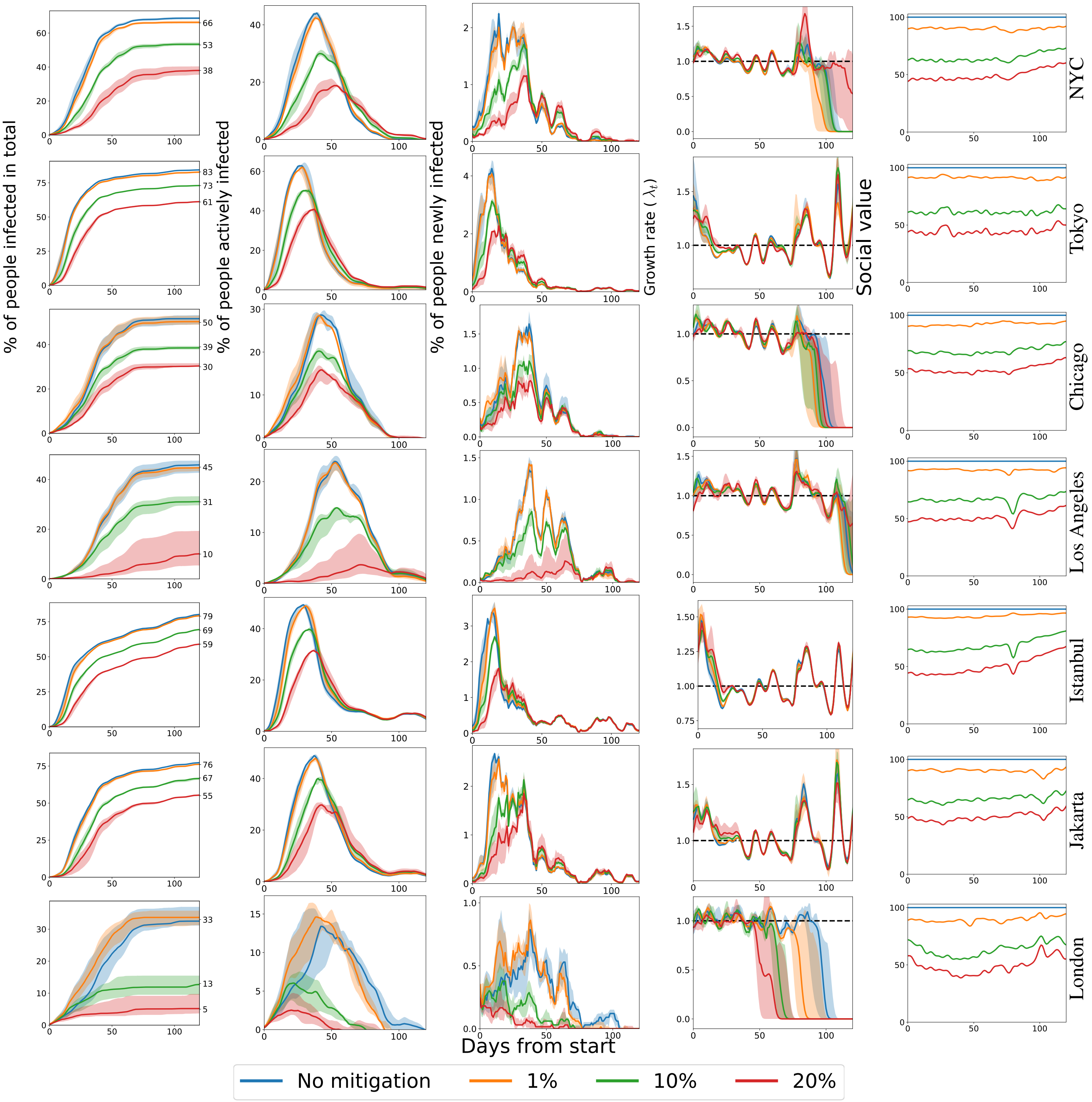

Figure S10. Infection spreading when protecting most active agents. The total number of infected people is reduced and the peaks of active infections are delayed and lowered with more active agents being protected.

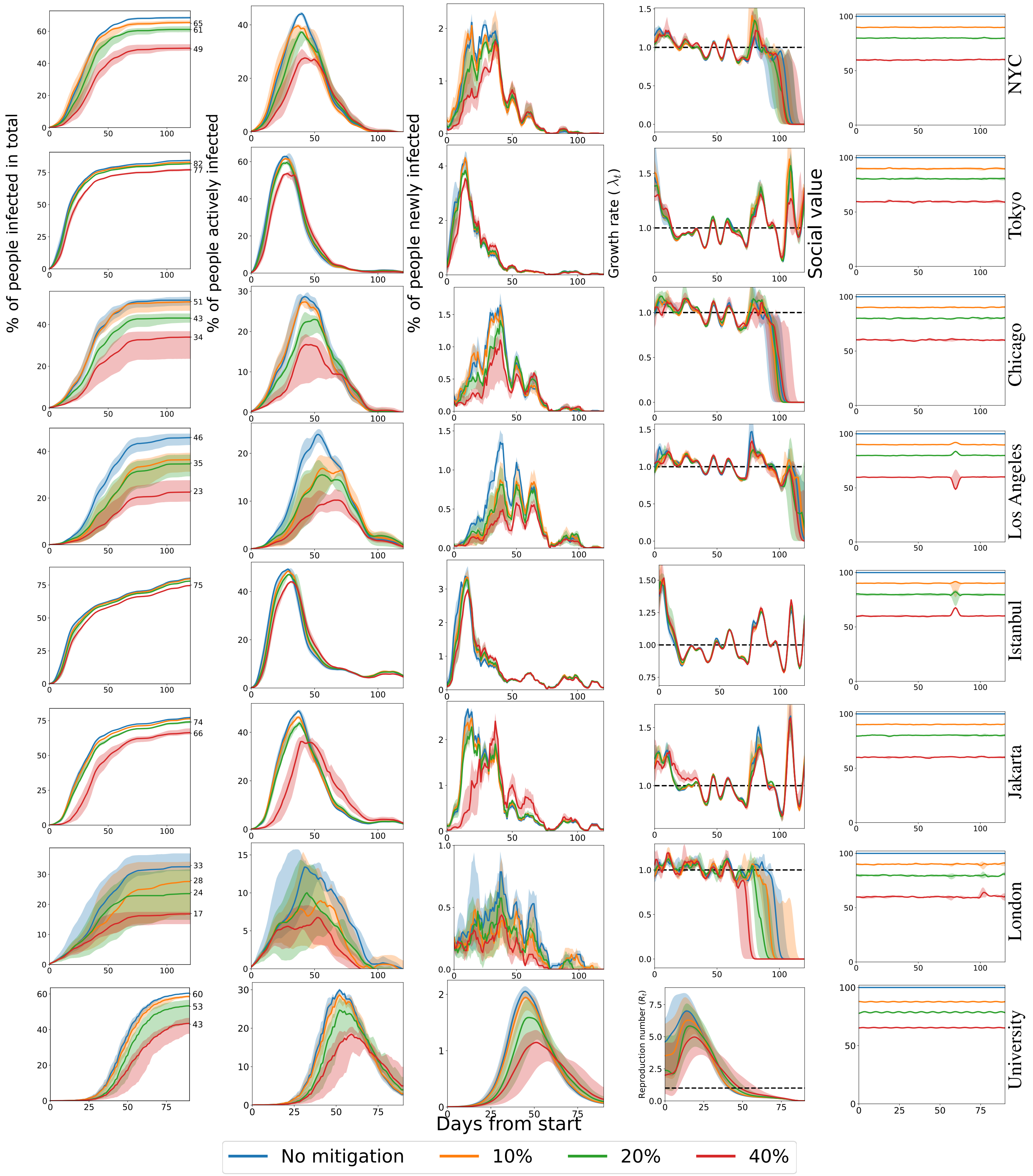

Figure S11. Infection spreading with the intervention strategy of closing random venues. Compared with closing popular venues, this strategy does not have a strong intervention effect. In some cities like Istanbul, when 40% venues are closed. the total number of infected people is reduced a little, less than 10%, but the social value is reduced by about 40%.

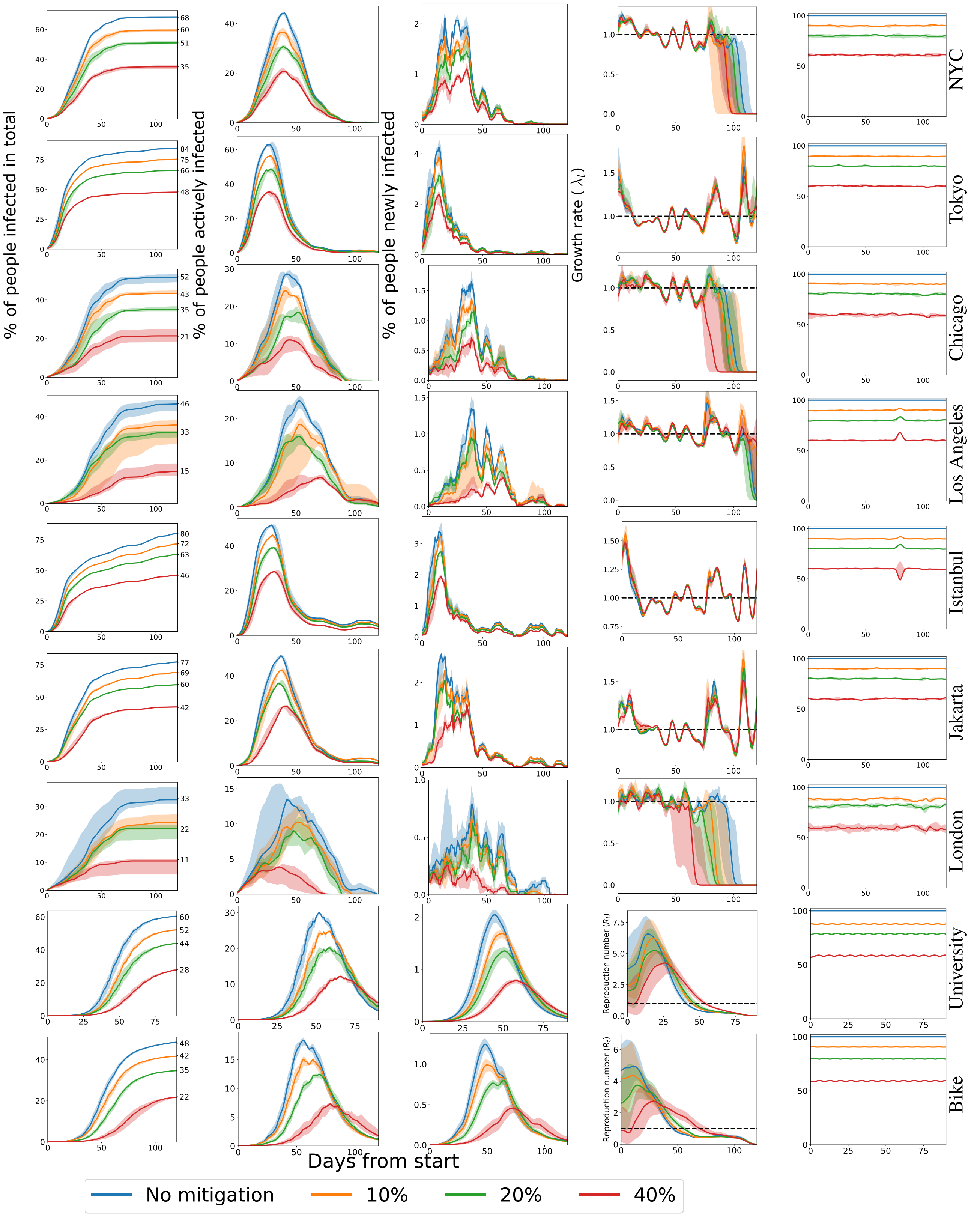

Figure S12. Infection spreading with the intervention strategy of protecting random people. Compared with protecting most active people, this strategy needs to protect more people to achieve the similar health value. When 40% people are protected, the total number of people infected can be reduced by about 20%, and the social value is reduced by 40%.

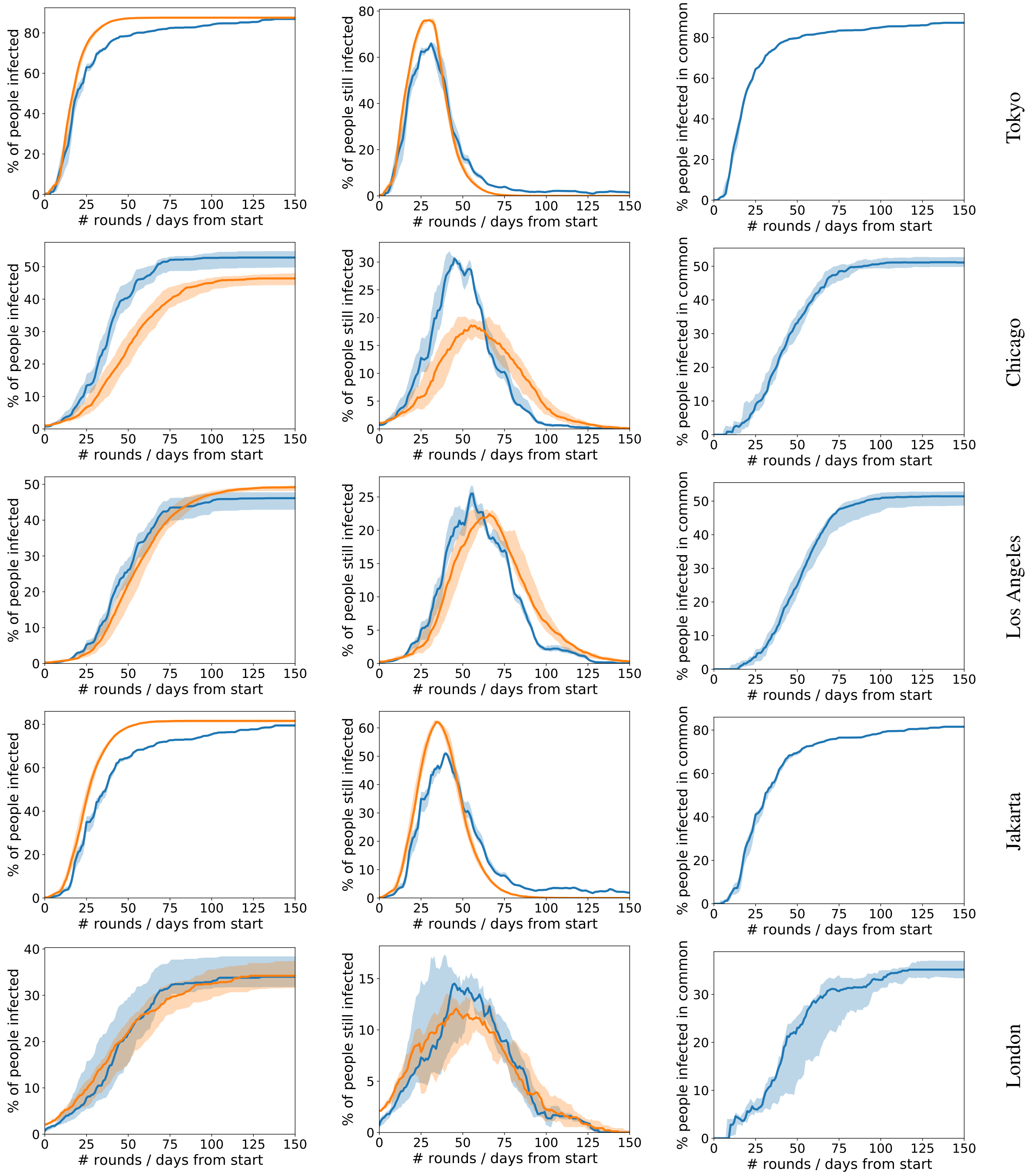

Figure S13. Infection spreading between mobility network and bipartite social networks. The infection curves between the two models are matched and the infection people in common also have a large percentage.

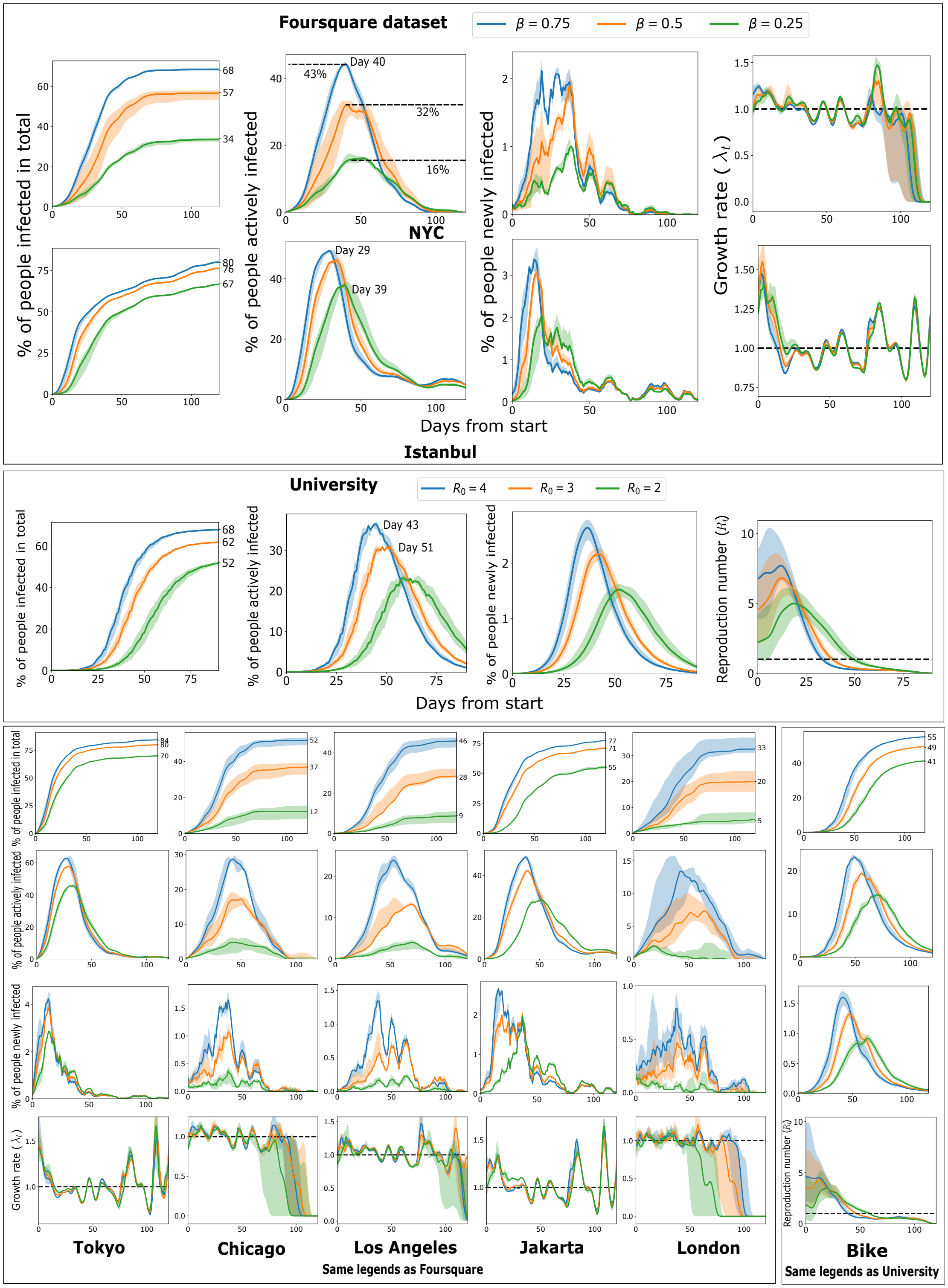

Figure S14. Infection spreading with varied transmission probabilities. In the Foursquare datasets, the transmission probabilities are provided directly. In the University and Bike datasets, the transmission probabilities are translated from R_0. With higher transmission probability values, the total number of people infected increase, and the peak of active cases get higher.

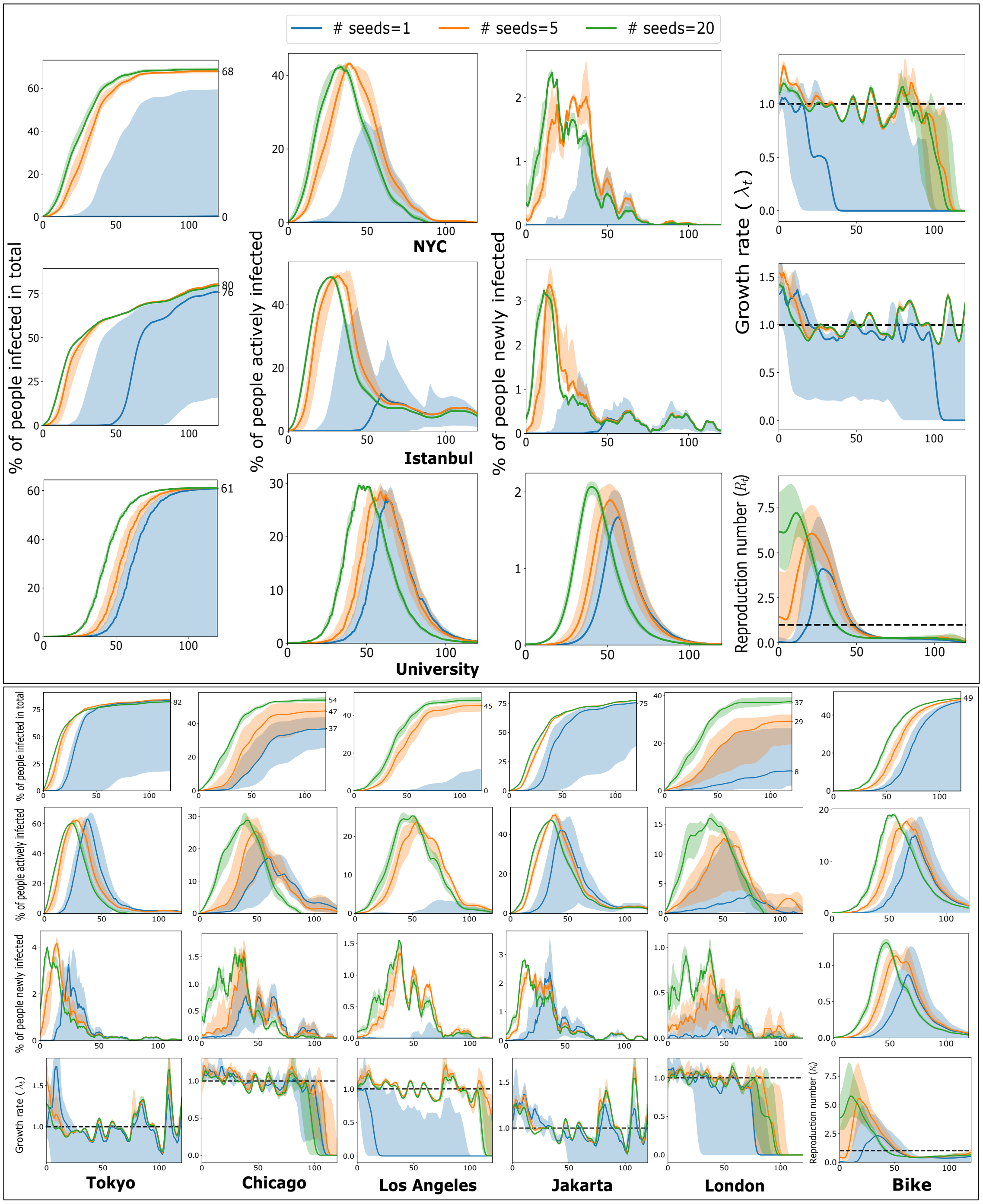

Figure S15. Infection spreading with a varied number of seeds. With a small number of initial seeds, the variance of infection is very large (shaded area). In most cases, when the number of seeds is enough, the total infection numbers and the peaks of people infected actively do not have a large difference.

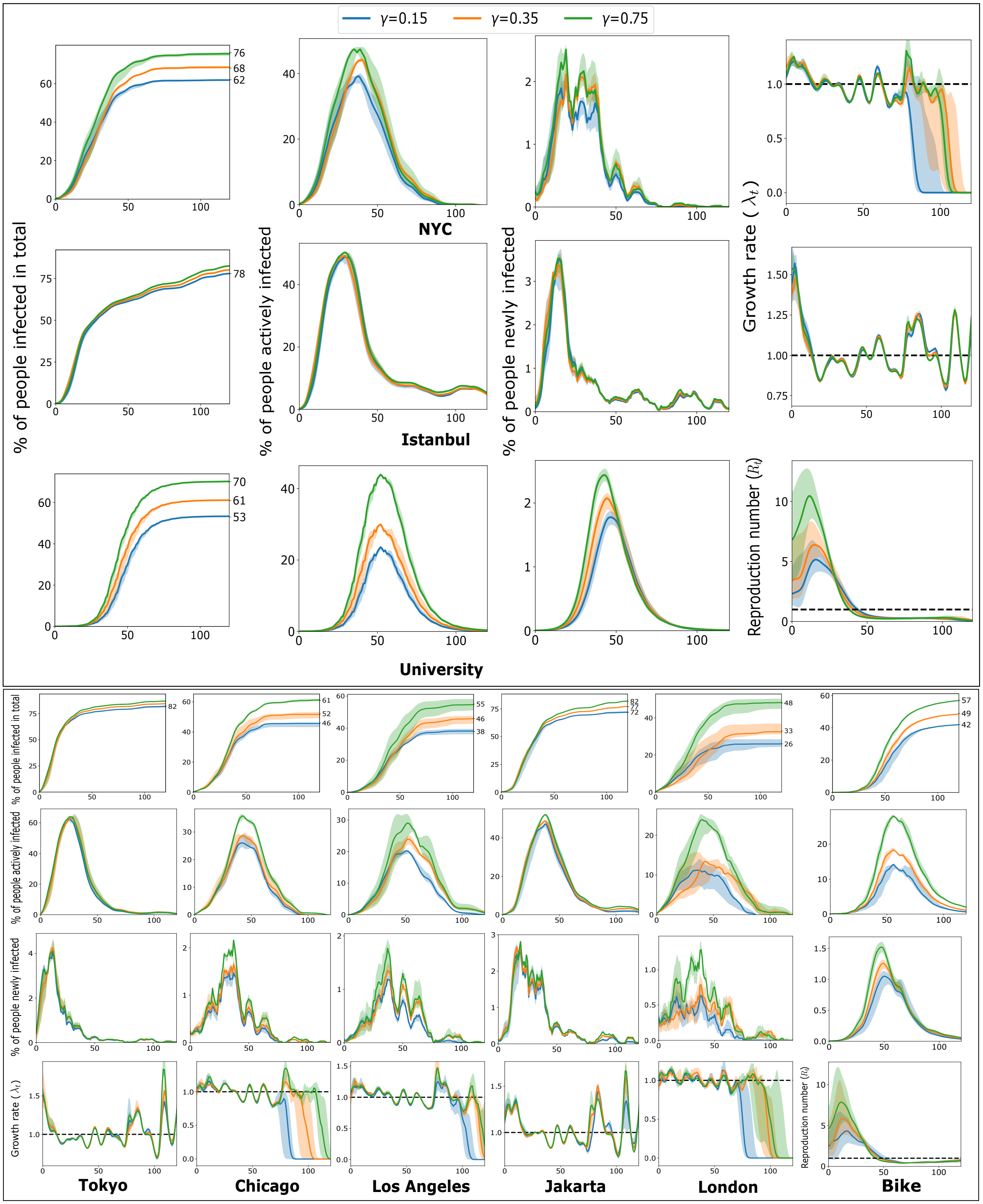

Figure S16. Infection spreading with the varying probabilities of being asymptomatic. With a high asymptomatic probability, there will be more infected people who have a longer duration to infect other susceptible people. It leads to a high percentage of people infected in total and a higher peak of people infected actively.

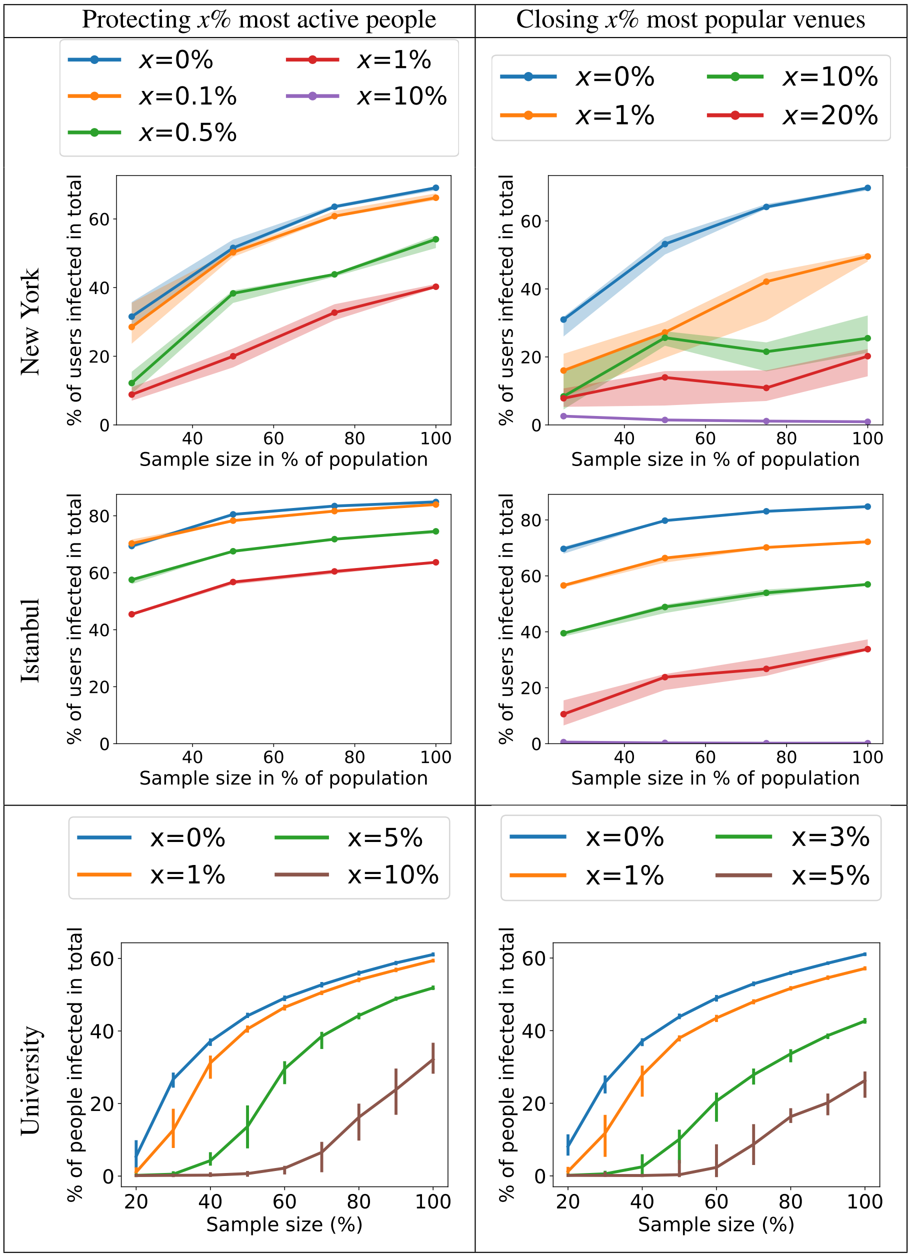

Figure S17. Infection spreading with the intervention strategy for different sizes of the dataset. The sample size is in the percentage of agents sampled. Using the intervention strategies to protect the most active people and close the most popular venues, the percent of infected agents decreases with sample size.

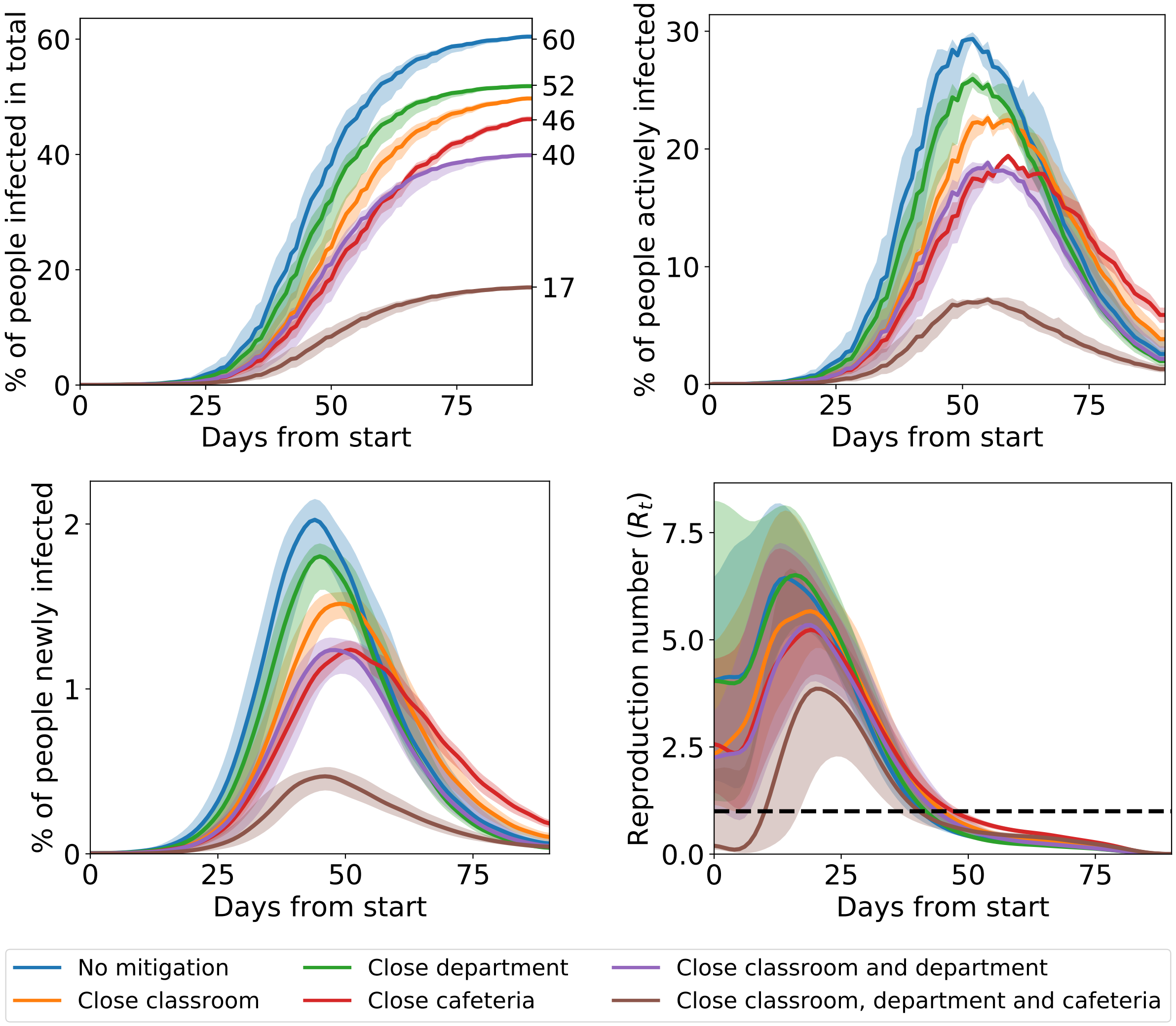

Figure S18. Closing some types of venues and constraining some activities are good intervention strategies in universities. In the university environment, there are many gathering events, especially taking courses and eating. In reality, many universities allow students to stay in the campus, but move the courses online and require students to take the meal back to dorms. So we test the spread in the university if some types of venues are closed. It shows that closing one type of venue is not enough to control the spreading. Only when we close all the classrooms and cafeteria, the total infection can be controlled under 20%, because students can also contact each other in other venues, due to the limited number of venues.
